# Supplementary material for: Dynamic tuneable G protein-coupled receptor monomer-dimer populations
Source: Nat Commun. 2018 Apr 27;9:1710. doi: 10.1038/s41467-018-03727-6 (PMC5923235; doi:10.1038/s41467-018-03727-6)
Supplement: Supplementary file 1 — Supplementary Information(PDF 2920 kb) [file 41467_2018_3727_MOESM1_ESM.pdf]

Supplementary information

Dynamic tuneable G protein-coupled receptor monomer-dimer populations.

Patricia M. Dijkman[1,5§], Oliver K. Castell[2,3§], Alan D. Goddard[1,6], Juan C. Munoz-Garcia[1,7], Chris de Graaf[4], Mark I. Wallace[2,8]* and Anthony Watts[1]*

[1]Biomembrane Structure Unit, Department of Biochemistry, University of Oxford, South Parks Road, Oxford OX1 3QU, UK

[2]Department of Chemistry, University of Oxford, South Parks Road, Oxford OX1 3QU, UK

[3]School of Pharmacy and Pharmaceutical Sciences, College of Biomedical and Life Sciences, Cardiff University, King Edward VII Avenue, Cardiff CF10 3NB, UK

[4]Division of Medicinal Chemistry, Faculty of Sciences, Amsterdam Institute for Molecules, Medicines and Systems (AIMMS), Vrije Universiteit Amsterdam, De Boelelaan 1108, 1081 HZ Amsterdam, The Netherlands

[5]Present address: Max Planck Institute for Biophysics, Max-von-Laue-Straße 3, 60438 Frankfurt am Main, Germany

[6]Present address: School of Life and Health Sciences, Aston University, Aston Triangle, Birmingham, B4 7ET, UK

[7]Present address: School of Pharmacy, University of East Anglia, Norwich Research Park, Norwich, NR4 7TJ, UK

[8]Present address: Department of Chemistry, King's College London, Britannia House, 7 Trinity Street, London SE1 1DB, UK

[§]These authors contributed equally to this work

*Correspondence and requests for materials should be addressed to A.W. (email: anthony.watts@bioch.ox.ac.uk) or to M.I.W. (email: mark.wallace@kcl.ac.uk)

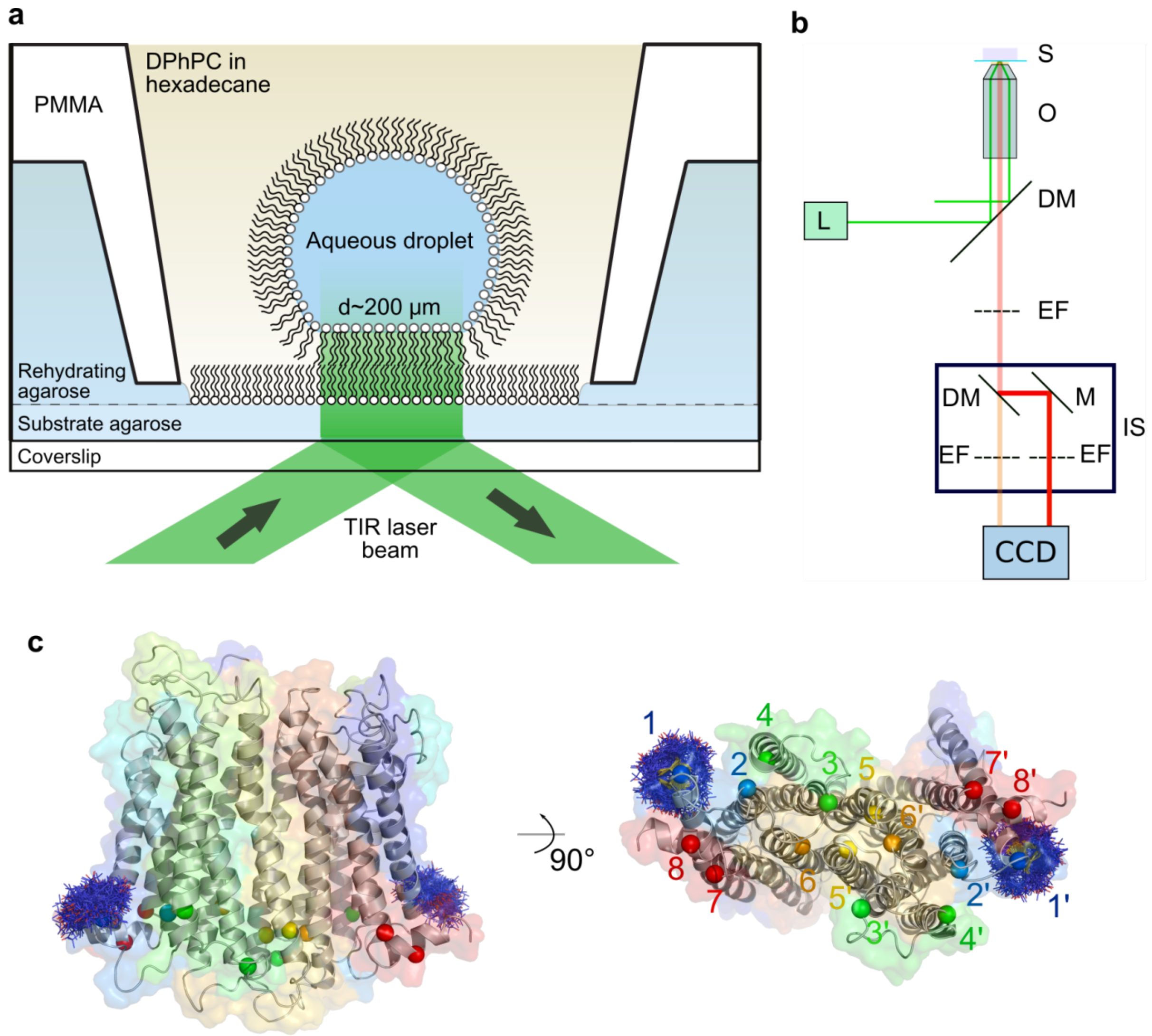

Supplementary Figure 1 - Experimental setup. (a) Schematic of a droplet-interface bilayer (DIB) for NST1 dimerisation measurements. Briefly, a bilayer is formed following the contacting of two lipid monolayers self-assembled at water-oil interfaces of an aqueous droplet and hydrogel surface. The bilayer is imaged by total-internal reflection fluorescence (TIRF) microscopy. More details are given by Leptihn et al.[1] **(b)** Optical setup of the TIRF microscope. The laser beam (L) at the donor wavelength is focussed on the sample (S) through the objective (O), using a dichroic mirror (DM). The emitted fluorescence passes the DM and an emission filter (EF), after which it is split by a second DM, separating the emission from the donor (Cy3) and acceptor (Cy5). **(c)** Single cysteine mutations were introduced into a Cys-depleted background mutant of rat NTS1 (see Methods) for site-directed labelling with either fluorescent dyes or spin labels for FRET and DEER experiments, respectively. The label sites are indicated on a model of a TM5-6 dimer interface: A90C$^{1.58}$ (TM1, blue), Y104C$^{2.41}$ (TM2, light blue), C172$^{3.55}$ (TM3, bright green, native Cys, reintroduced into the cysteine-depleted background mutant where it is mutated into a Ser), T186C$^{4.42}$ (TM4, green), A261C$^{5.52}$ (TM5, yellow), V307C$^{6.34}$ (TM6, orange), L371C$^{7.55}$ (TM7, red), and Q378C$^{8.52}$ (H8 dark red). Cα shown as spheres, and sites are numbered according to their helix position, with an apostrophe for the sites on the second protomer. Spin label rotamers calculated by MMM are shown for the TM1 label site.[2]

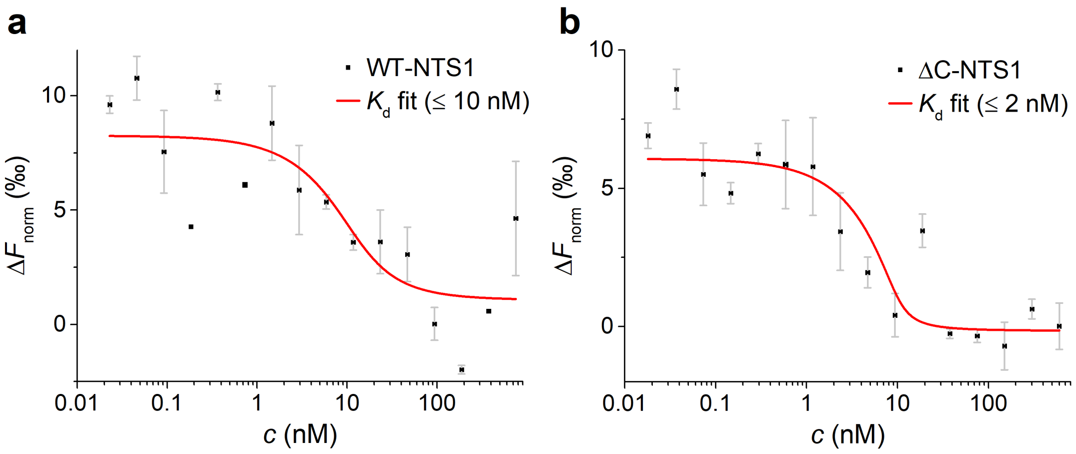

Supplementary Figure 2 - ΔC-NTS1 NT binding affinity compared to wild-type. Binding affinity of the cysteine depleted mutant of NTS1, ΔC-NTS1, was tested by microscale thermophoresis (MST) in detergent; 10 nM NT-Cy5 was added to a dilution series of **(a)** wild-type (WT) or **(b)** ΔC-NTS1 (0.02-750 and 0.02-600 nM, respectively), in duplicate for WT and triplicate for mutant NTS1 (error bars represent standard error). Control with denatured protein showed no binding (not shown). Fitted dissociation constants (K_d) are shown. Similar K_d values for NTS1-NT binding were previously determined by MST.[3]

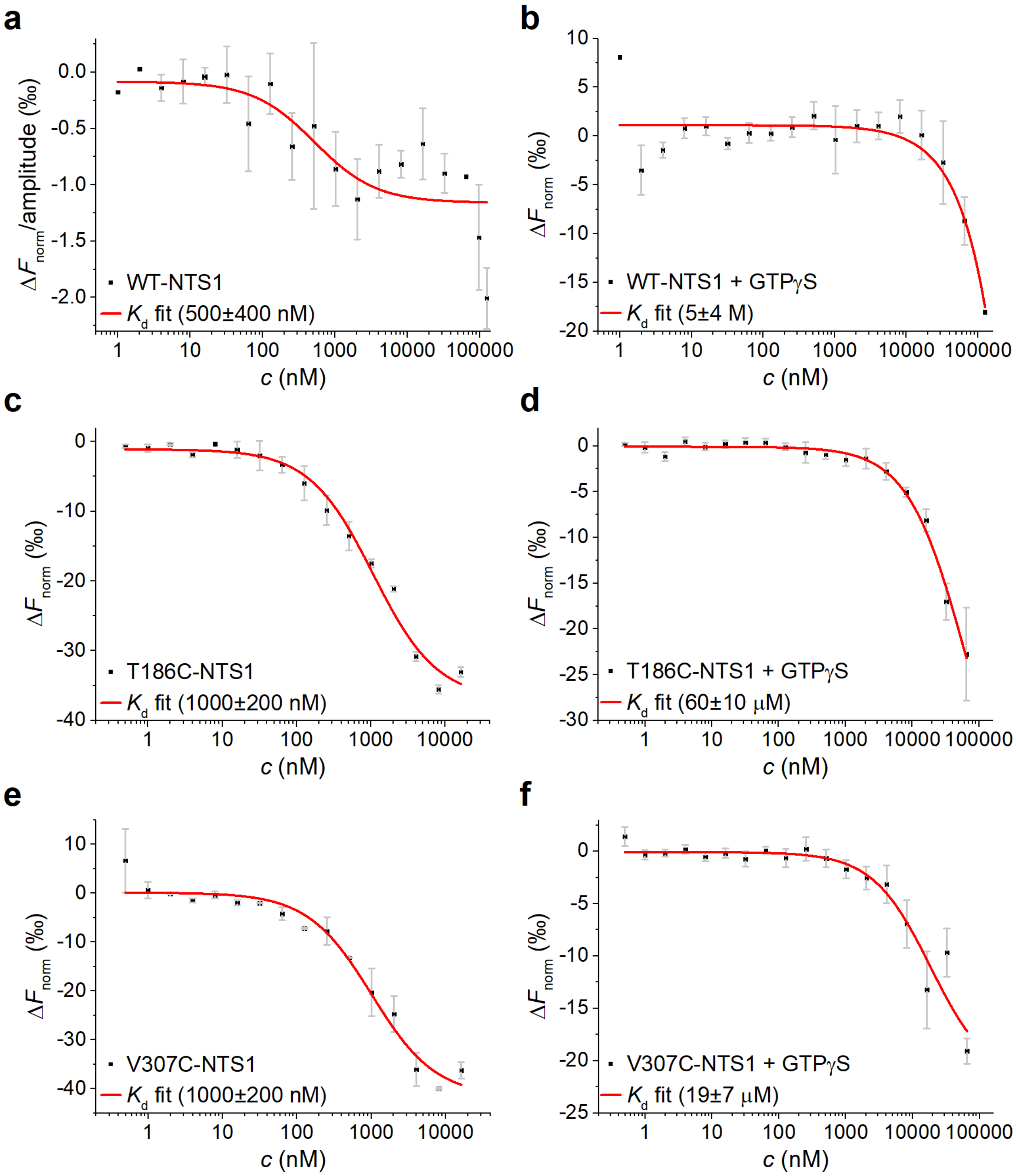

Supplementary Figure 3 – Cys-mutant NTS1 Gαi1 binding affinity compared to wild-type. G protein binding affinity of **(a,b)** WT-NTS1, **(c,d)** T186C$^{4.42}$, and **(e,f)** V307C$^{6.34}$ labelled with Alexa Flour 488 was tested by MST in detergent; 40 nM of labelled NTS1 was incubated with a dilution series of Gαi1 (0.5 nM to 62.5 μM), in the presence of 5 μM $NT_{8\text{-}13}$ **(a,c,e)**, or 5 μM $NT_{8\text{-}13}$ and 0.5 mM GTPγS **(b,d,f)**, as a negative control. Error bars represent standard errors of at least two experiments (*n* = 8 for WT+NT, *n* = 6 for WT+GTPγS, *n* = 2 for T186C+NT, *n* = 5 for T186C+GTPγS, *n* = 2 for V307C+NT, and *n* = 5 for V307C+GTPγS). Fitted dissociation constants (K_d) are shown.

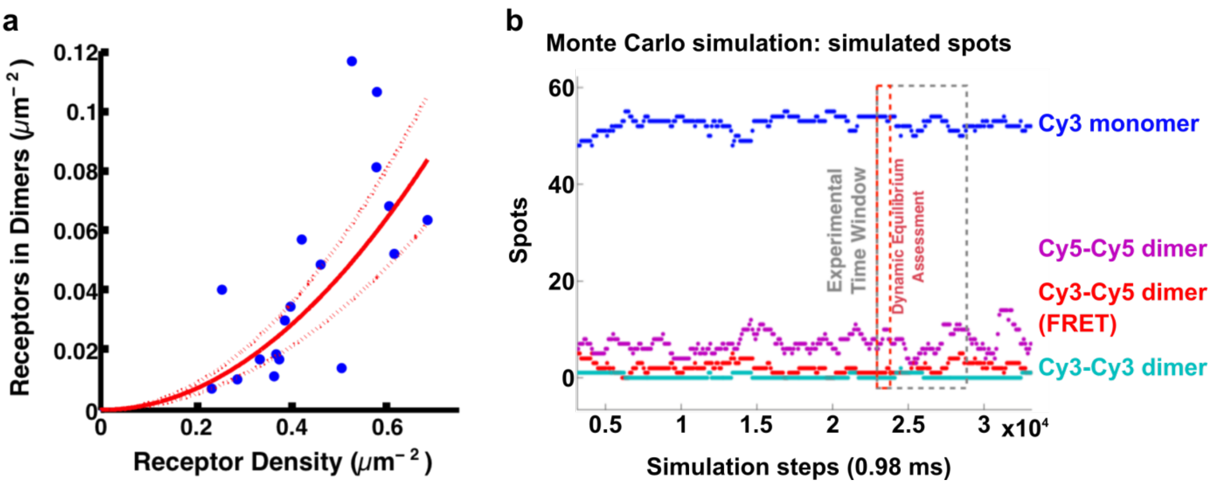

Supplementary Figure 4 - Variance in dynamic monomer-dimer equilibrium. **(a)** Experimental data: smFRET provides a time-limited glimpse into the dynamic equilibrium of donor and acceptor (FRET) species. Equilibrium number of detected donor and acceptor species in each experimental video are used to infer total number of monomeric and dimeric receptors in each experimental video (blue spots). Fitted line (x^2/a, a = 5.589, 95% CI [4.161,7.017], r^2 = 0.55) with 95% confidence bounds. Variation arises both as a result of variation in reconstituted receptor density and sampling of stochastic variance of single-molecule events. **(b)** At receptor numbers and density comparable to single-molecule imaging, Monte Carlo simulation reveals dynamic fluctuations in monomer and dimer species. The limited time sampled by single-molecule microscopy observation (grey dashed box) and assessment of equilibrium donor and acceptor species prior to significant photobleaching (red dashed box) illustrates how temporal variance in donor and acceptor spot detections can contribute to measured variation in experimental videos, as observed in **(a)**. See also Supplementary Movie 4 for illustrative Monte Carlo simulation at single-molecule receptor density.

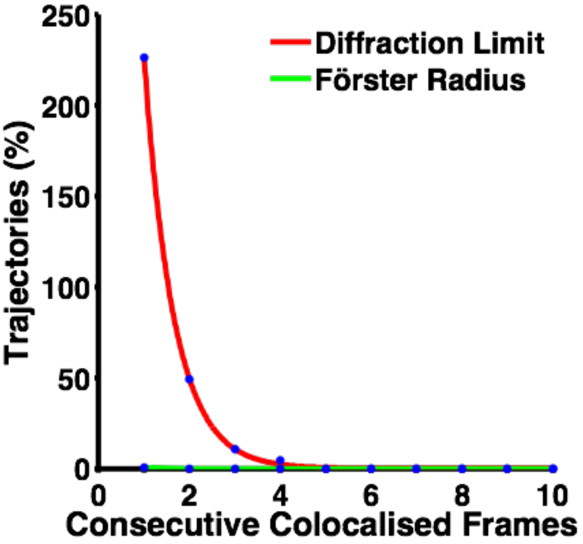

Supplementary Figure 5 - False positive single-molecule dimer detections are negligible via FRET. Coincident Cy3-NTS1 trajectories across multiple experimentally measured videos at 200 nm proximity radius (red, approximating to the proximity limit for determining diffraction-limited co-localisation) and 5 nm proximity radius (green, Förster radius of FRET detection) defining chance co-localisation. Measured co-localisation is expressed as the equivalent false positive rate of measured FRET acceptor trajectories (3+ frames). Given that only measured acceptor trajectories of three or more frames are accepted to represent NTS1 dimers, it can be concluded that for diffraction-limited detection of receptor-receptor interactions, a false positive rate of 15.1% would be expected. This is compared to 0.04% by single-molecule FRET as employed in this study.

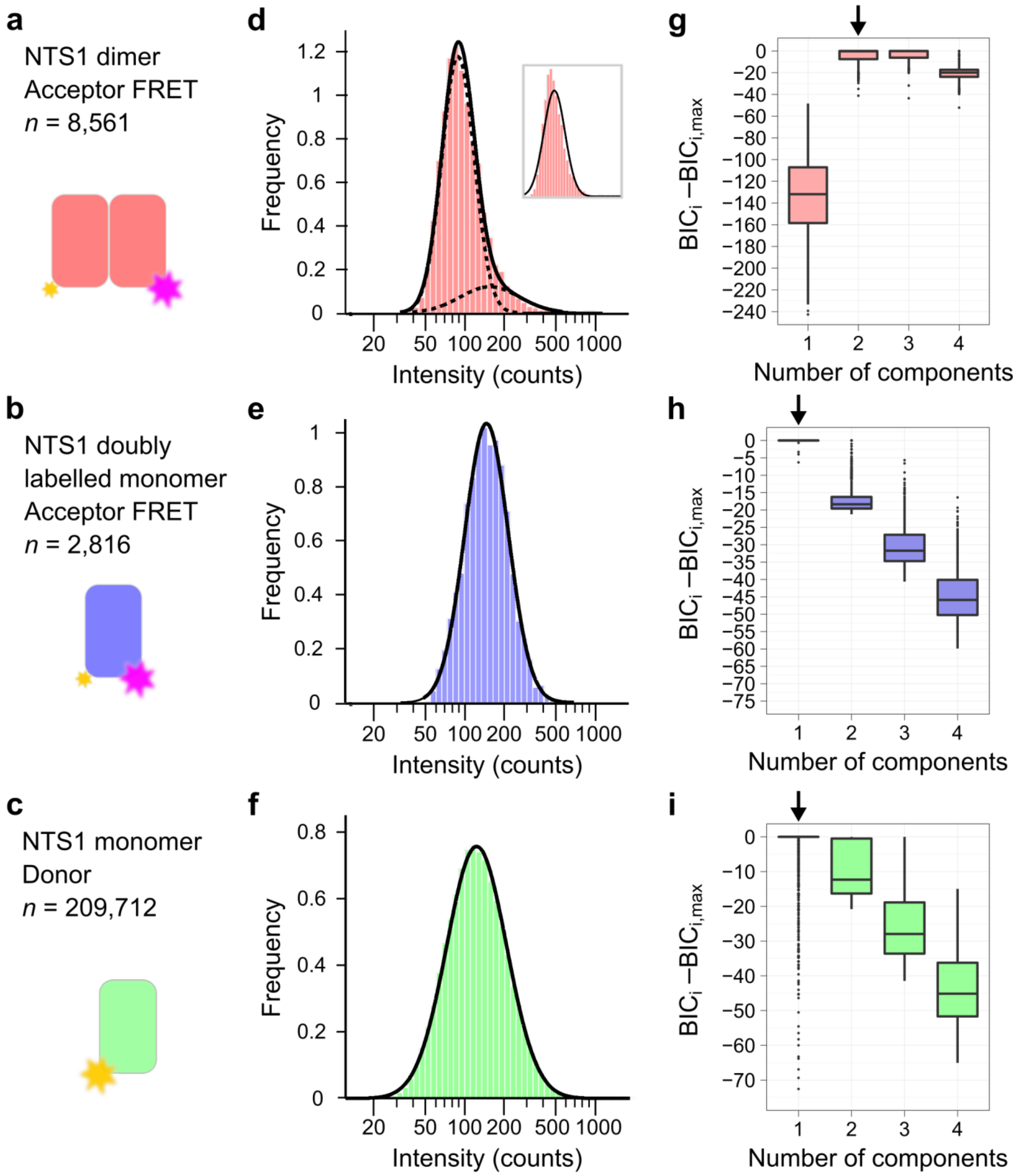

Supplementary Figure 6 – Likelihood analysis of intensity distribution models for single-molecule data. Gaussian mixture models with 1-4 components were fitted to single-molecule intensity data for **(a)** FRET acceptor intensity of dimeric species; **(b)** FRET acceptor intensity of doubly labelled, FRET capable, monomeric protein (control 1); and **(c)** direct donor excitation of monomeric singly labelled receptor (control 2). To account for differences in sample size between the intensity distributions of the three samples **(a-f)**, the data were bootstrapped 1,000 times, taking random samples of n = 1,000 for each of the three data sets. Four Gaussian mixture models composed of 1-4 components were fitted to each bootstrapped data sample, and the corresponding Bayesian information criterion (BIC) value was calculated to assess the relative likelihood of each of

the models. The highest BIC corresponds to the most probable model, and the absolute difference in BIC reflects the relative likelihood of the models, where differences larger than two can be seen as significant. To compare between bootstrapped samples, the BIC values for each sample were converted to BIC difference values by subtracting the largest BIC in the set (BIC_i-$BIC_{i,max}$). The resulting BIC values are summarised in the box plots shown in panels **(g-i)** for **(g)** FRET acceptor intensity of dimeric species; **(h)** FRET acceptor intensity of doubly labelled, FRET capable, monomeric protein (control 1); and **(i)** direct donor excitation of monomeric singly labelled receptor (control 2), respectively. The simplest model with highest BIC score on average over the bootstrapped samples is indicated with a black arrow in each case. The median difference in BIC values between the most probable and less probable models was more than 10 in each case, suggesting very strong evidence for the most probable models relative to the other models.

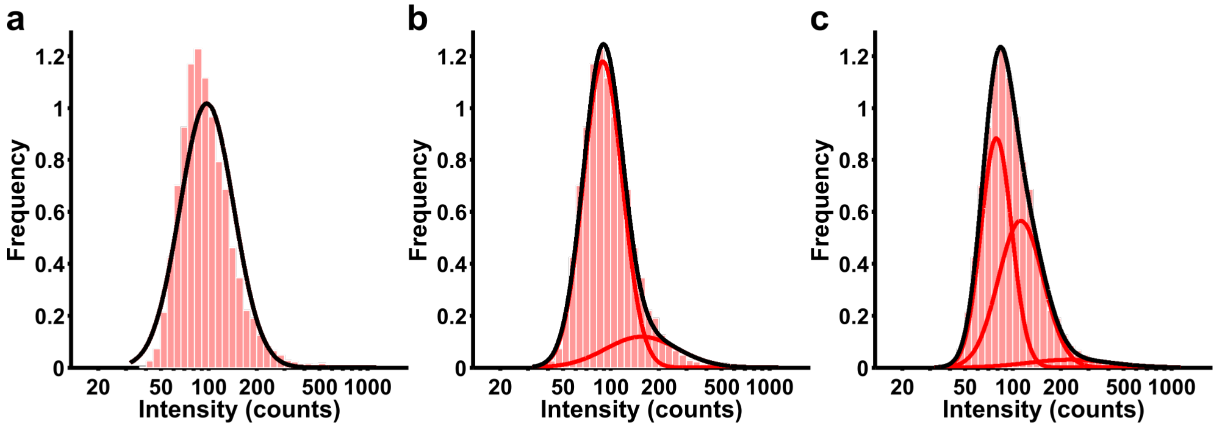

Supplementary Figure 7 – Gaussian mixture model fit to dimer acceptor single-molecule fluorescence intensity distribution. Gaussian mixture models with 1-4 components were fitted to the logarithm of the single-molecule intensity data (see also Supplementary Fig. 6). Here models composed of **(a)** one, **(b)** two, and **(c)** three components fit to FRET acceptor intensity of dimeric species are shown. Model likelihood analysis based on BIC values for the different models (Supplementary Fig. 6g) suggested a two or three component fit described the data substantially better than a single component.

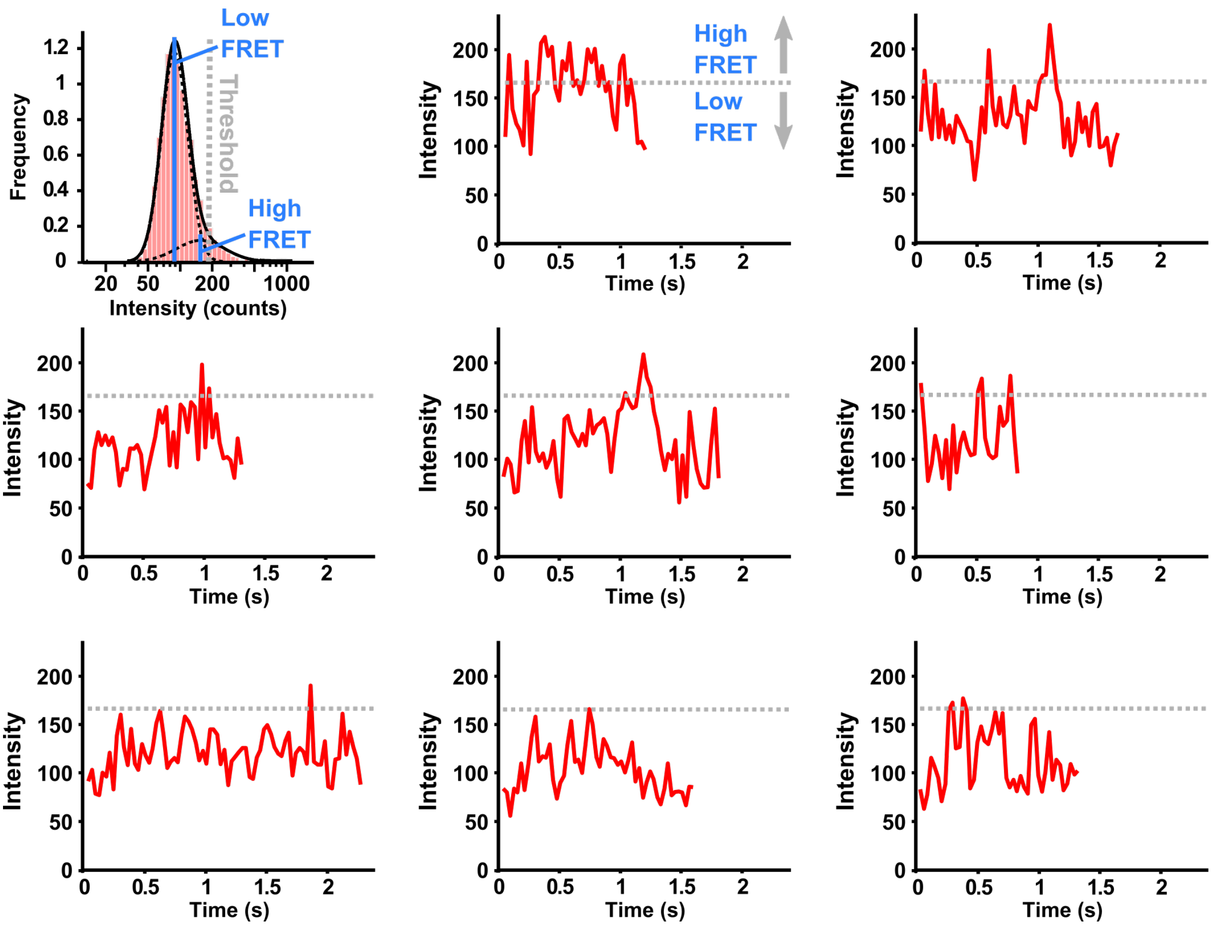

Supplementary Figure 8 - Additional representative single-molecule FRET acceptor intensity traces to accompany Fig. 4. A bimodal acceptor (Cy5) fluorescence intensity distribution, showing high- and low-FRET states, was measured for NTS1 Cy3-Cy5 dimers in single-molecule experiments (Fig. 2b and top left panel). A conservative threshold was defined (166.5 counts - grey dashed line), above which intensities have <1% probability of belonging to the low-FRET state population, and acceptor intensity was observed to cross this threshold, suggesting interconversion between the distinguishable low- and high-FRET dimer configurations. Representative NTS1 acceptor fluorescence intensity trajectories over time are shown here in addition to that shown in Fig. 4d.

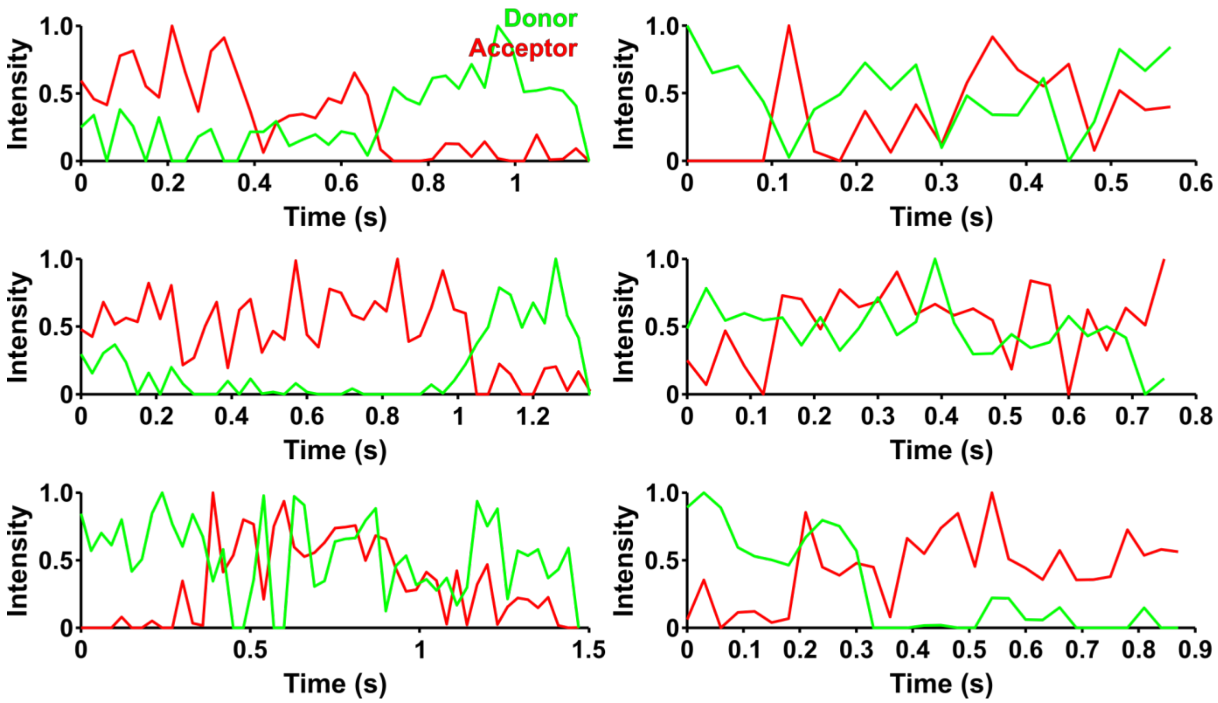

Supplementary Figure 9 - Additional representative single-molecule FRET NTS1 dimer fluorescence traces to accompany Fig. 4. NTS1 dimer fluorescence intensity trajectories over time for correlated donor (green: Cy3) and acceptor (red: Cy5) spots illustrate temporal fluctuation within a single dimer trajectory. Representative NTS1 acceptor fluorescence intensity trajectories over time are shown here in addition to that shown in Fig. 4b.

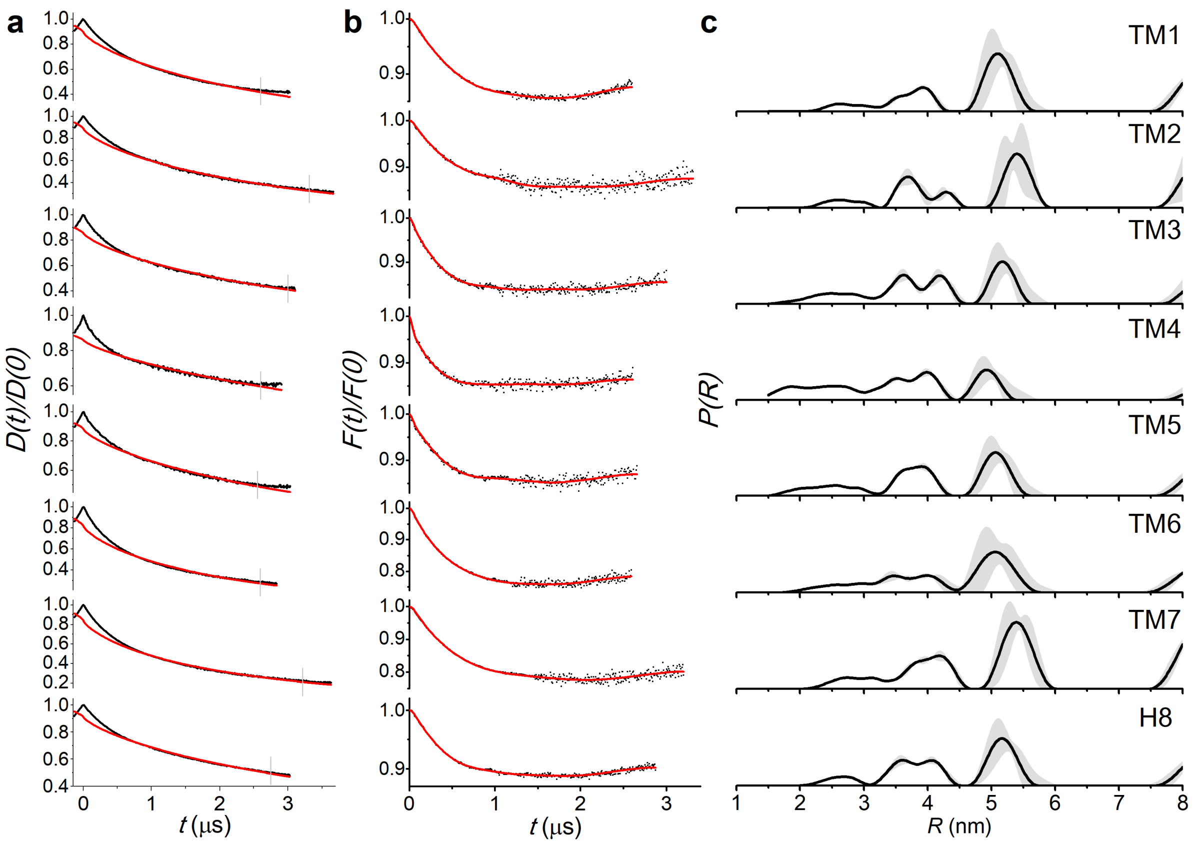

Supplementary Figure 10 – Inter-protomer DEER. DEER traces were recorded at Q-band for NTS1 samples spin labelled with MTSL at the intracellular side of TM1-7 and H8, and reconstituted into brain polar lipid liposomes. **(a)** Recorded dipolar evolution functions (black) and fitted stretched exponential backgrounds (*d* = 2.3, red) are shown. Grey vertical lines indicate the cut-off point after which data was discarded (to eliminate upturn, most likely caused by pulse overlap). **(b)** The background-corrected dipolar evolution functions (black data points) and fits thereof (red line), as well as **(c)** the corresponding distance distributions are also shown. Grey shaded areas in the distance distributions correspond to the estimated error (two times the standard deviation) for the probability for each of the distances in the distribution, covering the mean value of the probability plus/minus two times its standard deviation (determined by varying the background correction starting time in the DeerAnalysis validation tool).[4]

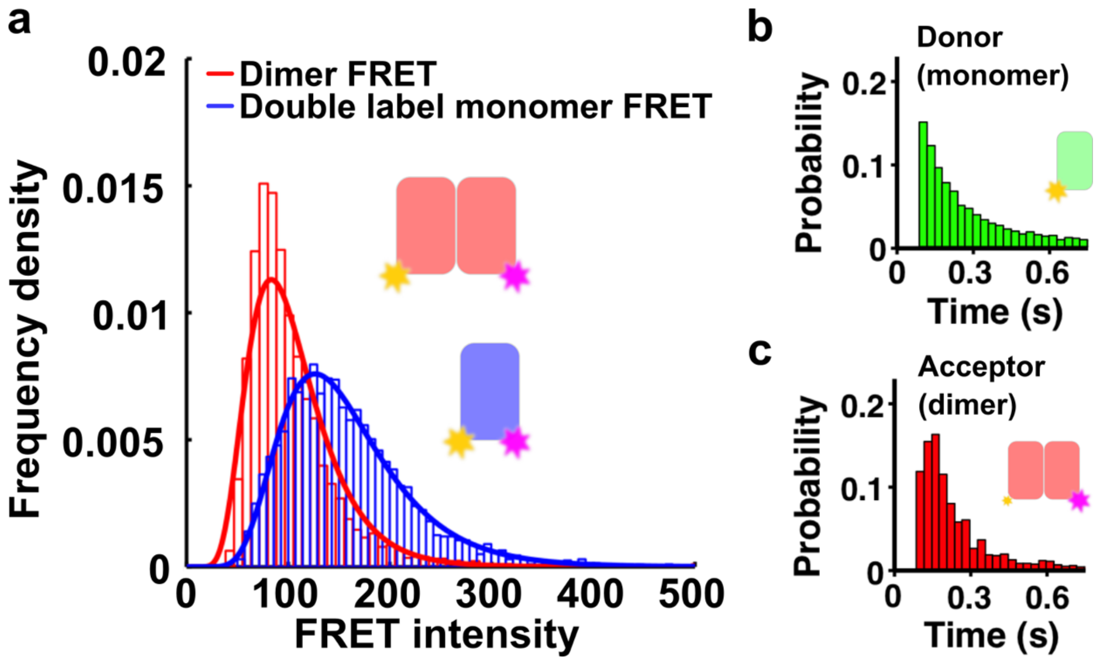

Supplementary Figure 11 - Characterisation of single-molecule intensity and trajectory lifetime distribution. (a) Acceptor intensity from single-molecule FRET measurements on dimers and doubly labelled, FRET capable, monomers. A double Cys mutant of NTS1 (blue, A90C$^{1.58}$-T186C$^{4.42}$; Cα-Cα distance estimated from 4UBO crystal structure 1.5 nm) doubly labelled with Cy3 and Cy5 was used to estimate the average T186C-T186C inter-monomer distance observed in the dimer FRET experiments with singly labelled receptor (red), giving an approximate distance of ~5.0 nm. **(b,c)** Trajectory lifetime distributions were measured in dimerisation experiments for **(b)** single-molecule donor and **(c)** acceptor trajectories. A monotonically decreasing lifetime probability is observed for donor trajectories as anticipated for photobleaching-limited observations of predominantly monomeric species. Acceptor lifetime probability is observed to be non-monotonic, with a lifetime probability maximum at 150 ms, and apparent subsequent photobleaching-limited observation. This is consistent with both rapid and longer decay processes with superimposed photobleaching, which is consistent with the presence of multiple FRET capable dimer conformations displaying differing stabilities. (Bin width = 1 frame = 30 ms, *n* = 17,853 trajectories (donor) and 1,167 trajectories (acceptor)).

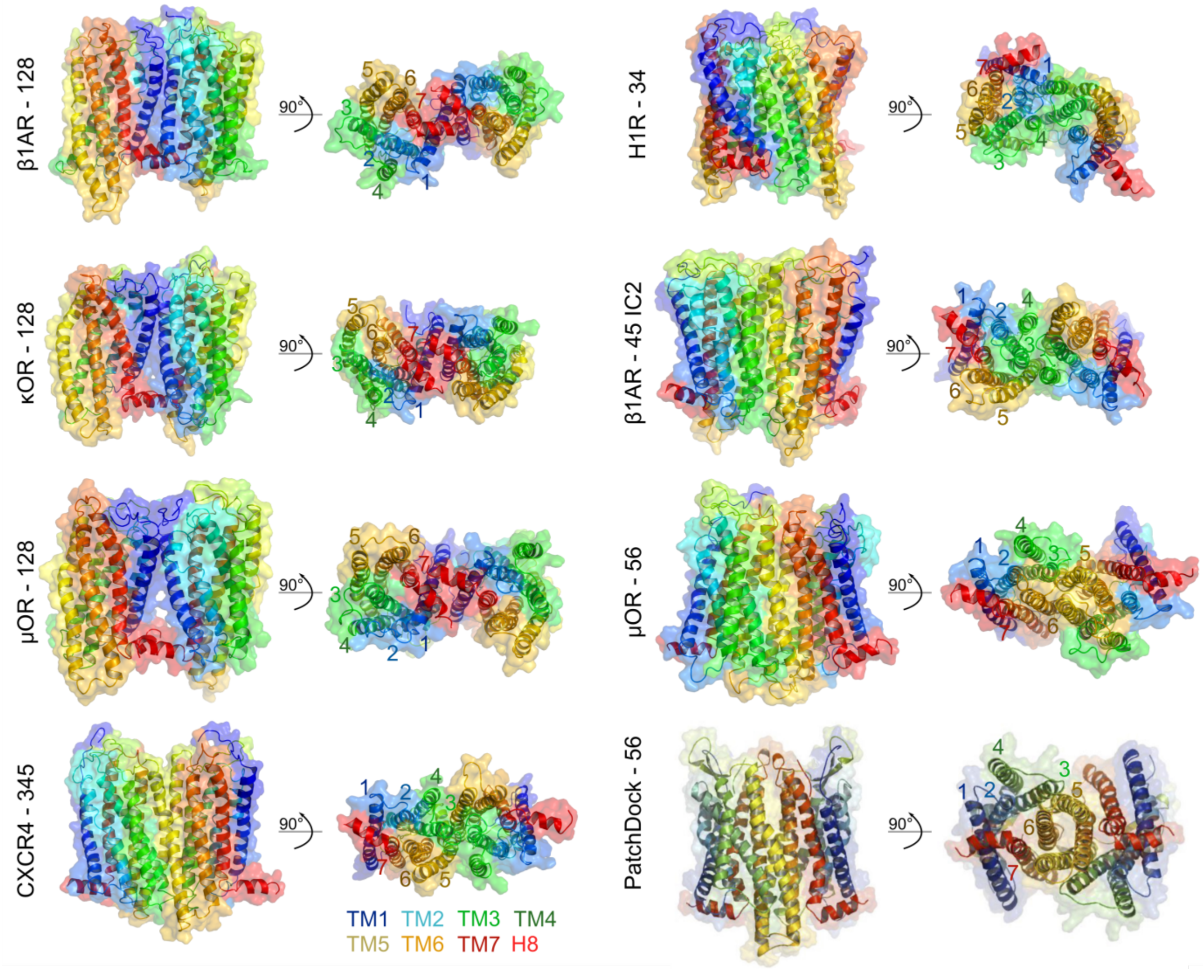

Supplementary Figure 12 - MD dimer models. Using a wild-type (wt-)NTS1 model built from the crystal structure of the NTS1 thermostabilised mutant (PDB code 4BUO, chain B[5]) back-mutated it to its native sequence, seven homology models of NTS1 dimers with different interfaces (cartoon and transparent surface are shown) were constructed based on the available crystal structures: $ß_1$ adrenergic (ß1AR, PDB 4GPO[6]), CXC chemokine type 4 (CXCR4, PDB 3OE0[7]), histamine 1 (H1R, PDB 3RZE[8]), κ-opioid (κOR, PDB 4DJH[9]) and μ-opioid (μOR, PDB 4DKL[10]) receptors The dimer models all appeared stable during 10 μs coarse-grain (CG) simulations in BPL-like lipid bilayers (see Methods). The CG simulation frames were converted back to atomistic (snapshot at 10 μs is shown). Helices are consistently rainbow-coloured as per the legend. Additionally, for comparison, protein-protein docking based on shape complementarity was carried out using the PatchDock webserver (http://bioinfo3d.cs.tau.ac.il/PatchDock)[11,12] with the wt-NTS1 model as input structure; the best ranked docking solution is also shown here. The solution showed a TM5-TM6 dimerisation interface in a parallel arrangement, in agreement with the major interface obtained from our fit of theoretical to experimental FRET efficiencies, i.e. TM5-TM6 interface based on the μ-opioid receptor crystal structure (PDB 4DKL[10]). A protein backbone RMSD value of 3.4 Å was obtained for this docked pose compared to the μ-opioid receptor-based homology model.

Supplementary Table 1 - Class A GPCR dimerisation interfaces. A (non-exhaustive) list of structural elements of class A GPCRs previously reported to be implicated in oligomerisation is given as well as the techniques used in identifying these. Abbreviations: AFM, atomic force microscopy; coIP, co-immunoprecipitation; EC, extracellular loop; ECD, extracellular domain; IB, immunoblotting; IC, intracellular loop; x-linking, cross-linking; X-ray, X-ray crystallography.

Receptor	Implicated TM/H									Technique
	1	2	3	4	5	6	7	8	Other	
**β_1-adrenergic**	x	x						x		X-ray; x-linking; IB[6]
				x	x				+IC2	
**β_2-adrenergic**						x				TM peptide competition; coIP; IB[13]
								x		X-ray[14]
								x		FRET[15]
**Rhodopsin**				x	x					X-linking; SDS-PAGE; AFM; modelling[16]
				x	x					X-linking; IB[17]
**M3 muscarinic**			x						+EC2	Mutagenesis; coIP; IB[18]
**acetylcholine**	x				x			x		Mutagenesis; BRET[19]
					x				+IC3	X-linking; coIP; IB; modelling[20]
**Dopamine D_2**						x	x			TM peptide competition; IB[21]
				x					+other unidentified	Mutagenesis; IB[22]
				x						X-linking; coIP; IB[23]
				x		x				X-linking; IB[24]
	x			x						X-linking; IB; RET; modelling[25]
**Dopamine D_3**	x	x						x		Mutagenesis; FRET[26]
				x	x					
**CCR5**	x									Mutagenesis; yeast-two-hybrid[27]
**chemokine**	x			x						Mutagenesis; TM peptide competition; coIP; FRET[28]
**CXCR4**			x	x	x					X-ray[7]
**chemokine**				x					+ cholesterol	TM peptide competition; cholesterol depletion; FRET[29]
**Bradykinin B_2**									ECD	Mutagenesis; IB[30]
									N-terminus (not EC)	TM peptide competition; x-linking; IB[31]
**Histamine H_1**	x		x	x						X-ray[8]
**α-factor**	x	x								Mutagenesis; FRET[32]
	x			x						X-linking; IB[33]
									N-terminus	X-linking; IB[34]
**δ-opioid**									C-terminus	Mutagenesis; IB[35]
**κ-opioid**	x	x						x		X-ray[9]
**μ-opioid**	x	x						x		X-ray[10]
					x	x				
**NTS1**	x	x		x						Computational modelling[36]
**Serotonin $5HT_{2C}$**	x			x	x					X-linking; IB[37]
**Adenosine A_{2A}**					x					Mutagenesis; TM peptide study; FRET; IB[38]
**Adenosine A_{1B}**				x						Mutagenesis; FRET; IB[39]
**BLT1 leukotrine**						x				TM peptide competition; mutagenesis; x-linking; SDS-PAGE; analytical gel filtration[40]

Supplementary Table 2 - Statistical analysis of the ensemble FRET efficiencies. The difference between the corrected mean apparent FRET efficiencies (E_{cor}) for all possible pairs of mutants in the absence (top) and presence (bottom) of neurotensin agonist is analysed by Bayesian methods to calculate the probability that the differences between sites are non-zero, or in other words that the likelihood of finding the same FRET efficiency for both sites is small, and thus that the sites can be reasonably said to have differing FRET efficiencies. Probabilities for a particular mutant (row) to have E_{cor} bigger (>, red) or smaller (<, blue) than another mutant (column) can be binned as being <0.683 (bellow one standard deviation, σ, grey), and thus not likely distinguishable, or at least 0.683-0.955 (σ light coloured text), 0.955-0.997 (2σ, dark coloured text), or >0.997 (3σ, darkest coloured text), and thus reasonably distinguishable.

TM/H (Residue)	1	2	3	4	5	6	7	8
E_{cor}	0.73 ±0.03	0.9 ±0.1	1.03 ±0.05	0.77 ±0.03	0.98 ±0.06	0.99 ±0.04	0.68 ±0.07	0.89 ±0.02
**1 (A90$^{1.58}$C)**	-	<0.896	<0.992	<0.770	<0.990	<1.000	>0.722	<0.999
**2 (Y104$^{2.41}$C)**		-	<0.735	>0.855	<0.624	<0.681	>0.911	>0.591
**3 (S172$^{3.55}$C)**			-	>0.989	>0.684	>0.659	>0.988	>0.949
**4 (T186$^{4.42}$C)**				-	<0.981	<1.000	>0.83	<0.977
**5 (A261$^{5.52}$C)**					-	<0.573	>0.979	>0.86
**6 (V307$^{6.34}$C)**						-	>0.992	>0.977
**7 (L371$^{7.55}$C)**							-	<0.974
E_{cor}(+NT)	0.77 ±0.04	0.9 ±0.1	1.02 ±0.02	0.78 ±0.03	1.05 ±0.09	0.82 ±0.04	0.7 ±0.1	0.89 ±0.02
**1 (A90$^{1.58}$C)**	-	<0.758	<0.998	<0.566	<0.974	<0.774	>0.522	<0.978
**2 (Y104$^{2.41}$C)**		-	<0.856	>0.738	<0.832	>0.622	>0.703	<0.553
**3 (S172$^{3.55}$C)**			-	>0.998	<0.590	>0.993	>0.923	>0.993
**4 (T186$^{4.42}$C)**				-	<0.946	<0.743	>0.563	<0.977
**5 (A261$^{5.52}$C)**					-	>0.946	>0.909	>0.910
**6 (V307$^{6.34}$C)**						-	>0.669	<0.869
**7 (L371$^{7.55}$C)**							-	<0.808

Supplementary Table 3 – Likelihood analysis of dimer model fit to the ensemble FRET data. (See next page).

128			345	34	45	56	*n*	RSS	AIC	ΔAIC
**(β1AR)**	**(κOR)**	**(μOR)**	**(CXCR4)**	**(H1R)**	**(β1AR)**	**(μOR)**				
0.04				0.13		0.83	3	0.080	-9.2	3.2
		0.04		0.12		0.83	3	0.080	-9.2	3.3
				0.17		0.83	2	0.080	-11.3	1.2
		0.12				0.88	2	0.081	-10.8	1.6
0.11						0.89	2	0.081	-10.8	1.6
	0.09					0.91	2	0.083	-10.7	1.7
						1	1	0.085	-12.4	-
0.11			0.89				2	0.108	-9.3	3.1
		0.1	0.9				2	0.108	-9.3	3.2
	0.03		0.93	0.04			3	0.110	-7.3	5.1
	0.05		0.95				2	0.110	-9.2	3.2
			0.92	0.08			2	0.110	-9.3	3.1
			1				1	0.111	-11.2	1.2
0.13					0.87		2	0.120	-8.5	4.0
		0.11			0.89		2	0.121	-8.4	4.1
				0.13	0.87		2	0.123	-8.5	3.9
	0.06				0.94		2	0.123	-8.4	4.1
					1		1	0.124	-10.3	2.1
0.05				0.95			2	0.194	-4.3	8.1
				1			1	0.194	-6.3	6.1
0.57	0.43						2	0.313	0.5	13.0
0.78		0.22					2	0.316	0.7	13.1
1							1	0.318	0.7	13.1
	1						1	0.321	-1.3	11.2
		1					1	0.331	-0.9	11.5

+NT

128			345	34	45	56	*n*	RSS	AIC	ΔAIC
**(β1AR)**	**(κOR)**	**(μOR)**	**(CXCR4)**	**(H1R)**	**(β1AR)**	**(μOR)**				
		0.004		0.28		0.72	3	0.085	-9.2	2.8
				0.28		0.72	2	0.085	-11.2	0.8
		0.17				0.83	2	0.089	-10.2	1.9
		0.17	0.01			0.82	3	0.089	-8.2	3.9
0.14			0.09			0.77	3	0.092	-8.0	4.0
0.15						0.86	2	0.092	-9.9	2.1
	0.12		0.06			0.82	3	0.094	-7.9	4.1
	0.13					0.87	2	0.094	-9.9	2.2
			0.30			0.70	2	0.097	-9.8	2.2
					0.12	0.88	2	0.098	-9.5	2.5
						1	1	0.098	-11.4	0.7
		0.13	0.87				2	0.101	-10.2	1.8
0.08			0.84	0.08			3	0.102	-8.3	3.7
0.11			0.89				2	0.102	-10.2	1.9
			0.83	0.17			2	0.102	-10.5	1.6
	0.06		0.94				2	0.104	-10.1	2.0
			1.00				1	0.105	-12.1	-
		0.11		0.05	0.85		3	0.108	-7.8	4.3
		0.13			0.87		2	0.108	-9.7	2.4
0.08				0.11	0.82		3	0.109	-7.9	4.2
0.12					0.88		2	0.109	-9.7	2.4
				0.19	0.81		2	0.109	-10.1	2.0
					1.00		1	0.113	-11.6	0.4
				1			1	0.171	-7.8	4.3
0.44	0.17	0.39					3	0.305	2.3	14.4
0.47		0.53					2	0.305	0.4	12.4
0.50	0.51						2	0.306	0.3	12.3
	0.43	0.57					2	0.309	0.4	12.5
		1					1	0.310	-1.4	10.6
	1						1	0.312	-1.7	10.3
1							1	0.312	-1.5	10.6

(Continued) Supplementary Table 3 – Likelihood analysis of dimer model fit to the ensemble FRET data. Linear combinations of seven structural NTS1 dimer models (Supplementary Fig. 12) were compared to the experimental FRET results (for apo receptor, top half of the table, and in the presence of agonist, +NT, bottom half of the table). For each model the intradimer distance for each label site was measured along the CG simulation from which a theoretical FRET efficiency was calculated. The residual sum of squares (RSS) of the experimental FRET minus the theoretical FRET for the (linear combination of) model(s) was minimised by optimising the fractional contribution of each model (columns 1-7) to the composite linear combination (see Methods). The different combinations of models are ranked according to lowest RSS, excluding those combinations of models for which the fractional contribution of any additional models equalled zero. In addition, the Akaike information criterion (AIC) value for each model was calculated to assess the relative likelihood of each of the models, where the lowest AIC value (highlighted in green) indicates the most probable model and a difference of 2 in the AIC value is taken to be significant.[41] The ΔAIC column gives the difference in AIC between each model and the model with lowest AIC; ΔAIC values for models that are indistinguishable from the most probable model (lowest AIC), i.e. for which ΔAIC<2, are highlighted in red.

Supplementary Table 4 - Likelihood analysis of dimer model fit to the DEER data. For the three most likely NTS1 dimer models, based on comparison with the ensemble FRET data (Supplementary Table 3), theoretical DEER distance distributions were calculated (Fig. 5e) taking into account the contribution of the spin label rotamers (see Methods). Linear combinations of the theoretical distance distributions for the three models were compared to the experimental DEER distance distributions; the residual sum of squares (RSS) of the experimental minus the theoretical distance distributions for the (linear combination of *n*) model(s) was minimised by optimising the fractional contribution of each model (columns 1-3) to the composite linear combination (see Methods). The different combinations of models are ranked according to lowest RSS. In addition, the Akaike information criterion (AIC) value for each model was calculated to assess the relative likelihood of each of the models, where the lowest AIC value (highlighted in green) indicates the most probable model and a difference of 2 in the AIC value is taken to be significant.[41] The ΔAIC column gives the difference in AIC between each model and the model with lowest AIC.

**model contribution**						
**128**	**34**	**56**	***n***	**RSS**	**AIC**	**ΔAIC**
0.31	0.36	0.33	3	177.3	360.3	-
0.45	0.55		2	206.5	754.5	394.2
0.48		0.52	2	208.1	789.2	34.7
	0.53	0.47	2	210.9	814.5	25.3
	1		1	284.2	1602.5	788.0
		1	1	304.4	1767.9	165.4
1			1	321.3	1931.2	163.3

Supplementary Table 5 – Primers used to remove or introduce cysteines for site-directed labelling. Introduced mutation is highlighted in red.

Mutation	Primers
**C272S**	For: 5' GTG GAG CGC TAC TTG GCC ATC AGC CAT CCC TTC AAG GCC AAG ACC 3'<br>Rev: 5' GGT CTT GGC CTT GAA GGG ATG GCT GAT GGC CAA GTA GCG CTC CAC 3'
**C278S**	For: 5' GAG CAG GGC CGA GTG AGC ACC GTG GGC ACA CAC 3'<br>Rev: 5' GTG TGT GCC CAC GGT GCT CAC TCG GCC CTG CTC 3'
**C332S**	For: 5' CGA CGC CTG ATG TTC AGC TAT ATC TCG GAT GAA 3'<br>Rev: 5' G TTC ATC CGA GAT ATA GCT GAA CAT CAG GCG TCG 3'
**C386S**	For: 5' CTG TCC ACG CTG GCC AGC CTT TGT CCT GGG TGG 3'<br>Rev: 5' CCA CCC AGG ACA AAG GCT GGC CAG CGT GGA CAG 3'
**C388A**	For: 5' ACG CTG GCC AGC CTT GCG CCT GGG TGG CGC CAC 3'<br>Rev: 5' GCG GCG CCA CCC AGG CGC AAG GCT GGC CAG CGT 3'
**A90C**	For: 5' GCC TTC ACT CTA TGC CGG AAG AAG TCA CTG C 3'<br>Rev: 5' G CAG TGA CTT CTT CCG GCA TAG AGT GAA GGC 3'
**Y104C**	For: 5' CAG AGC ACT GTG CAT TGC CAC CTG GGC AGC CTG 3'<br>Rev: 5' CAG GCT GCC CAG GTG GCA ATG CAC AGT GCT CTG 3'
**S172C**	For: 5' CGC TAC TTG GCC ATC TGC CAT CCC TTC AAG GCC 3'<br>Rev: 5' GGC CTT GAA GGG ATG GCA GAT GGC CAA GTA GCG 3'
**T186C**	For: 5' TCC CGC AGC CGC TGC AAG AAA TTC ATC 3'<br>Rev: 5' GAT GAA TTT CTT GCA GCG GCT GCG GGA 3'
**A261C**	For: 5' CTA AAC ACC GTG ATT TGC AAC AAA CTG ACA GTC 3'<br>Rev: 5' GAC TGT CAG TTT GTT GCA AAT CAC GGT GTT TAG 3'
**V307C**	For: 5' GCC CTG CGC CAC GGA TGC CTC GTC TTA CGT GCT 3'<br>Rev: 5' AGC ACG TAA GAC GAG GCA TCC GTG GCG CAG GGC 3'
**L371C**	For: 5' CCC ATC CTC TAC AAC TGC GTC TCC GCC AAC TTC 3'<br>Rev: 5' GAA GTT GGC GGA GAC GCA GTT GTA GAG GAT GGG 3'
**Q378C**	For: 5' TCC GCC AAC TTC CGC TGC GTC TTT CTG TCC ACG 3'<br>Rev: 5' CGT GGA CAG AAA GAC GCA GCG GAA GTT GGC GGA 3'

Supplementary Table 6 - Estimation of lipid-to-protein ratio from sucrose density gradients. The density (ρ_{PL}) of proteoliposome samples prepared for FRET and DEER experiments was estimated by running the samples on a sucrose density gradient from which the partial specific volume ($\bar{\nu}_{\mathrm{PL}}$) of the proteoliposomes was calculated.[42] The lipid-to-protein ratio (L:P, w/w) was estimated using $\bar{\nu}_{\mathrm{lipids}}$=0.986 mL g^{-1} and $\bar{\nu}_{\mathrm{NTS1}}$=0.735 mL g^{-1}.[43] The weight ratio was converted to a molar ratio assuming an average molecular weight of 650 g mol^{-1} for BPL.

**Initial L:P (mol:mol)**	$\boldsymbol{\rho_{\mathrm{PL}}}$ **(kg dm^{-3})**	$\boldsymbol{\bar{\nu}_{\mathrm{PL}}}$ **(mL g^{-1})**	**Final L:P (w/w)**	**Final L:P (mol:mol)**	**Receptors per vesicle**	**Density (10^3 µm^{-2})**
**FRET** 6000:1	1.03±0.2	0.97±0.2	12±2	840±20	90±20	1.6±0.3
**DEER** 1500:1	1.05±0.2	0.95±0.2	6±1	410±10	180±40	3.1±0.6

Supplementary Note 1 - Equilibrium monomer-dimer distribution, rate of dimer formation, and calculation of kinetic parameters

The dynamic equilibrium monomer-dimer distribution was calculated from the initial two dimensional membrane density of donor and acceptor spots on initial frames of each video, prior to significant photobleaching. Frame one was discarded due to a triggering asynchrony between the laser shutter and camera resulting in under-illumination in the first frame. Trajectories existing within the next five frames were considered to represent the equilibrium visible donor and acceptor densities. Across 19 videos an average density of 0.072 and 0.0056 spots μm^{-2} was measured for visible monomer (donor) and dimer (acceptor) spots, respectively. The ratio of labelled species was then used to infer the total monomer and dimer species concentrations.

The probability of Cy3-Cy3 dimers is sufficiently low that we assume the total number of detectable Cy3-labelled receptors is represented by the total of equilibrium detected donor and acceptor spots, each comprising one Cy3-labelled receptor. With Cy3-labelled protein representing 0.182 of the total receptor species, total receptor density is approximated by equation (S1).

$$\text{Receptor density} = (A + D) \text{ x } (1/(\text{Cy3}/(\text{Cy3} + \text{Cy5} + U))) \qquad \text{(Eq. S1)}$$

Where A represents the visible equilibrium acceptor spot density, D represents the visible equilibrium donor acceptor spot density, with Cy3, Cy5, and U representing the relative proportions of Cy3-labelled, Cy5-labelled and unlabelled protein, respectively.

From the labelling ratio, the probability, and therefore relative proportions, of dimers belonging to all combinations of Cy3, Cy5, and unlabelled species was calculated. FRET-capable Cy3-Cy5 dimers represent 0.268 of the dimeric population. Consequently, the total monomer and dimer densities in our experiments are calculated as 0.386 and 0.021 (i.e. 0.042 receptors) μm^{-2}, respectively, at a total receptor density of 0.428 μm^{-2}.

Cy3-Cy3 dimers were noted to be particularly rare representing ~3% of dimers and therefore ~0.27% of all species. This corresponds to approximately 0.98% of donor channel spots prior to photobleaching, in keeping with the intensity distribution measured for donor spots being adequately

described by a single log-normal distribution (Fig. 2).

The detection of trajectories in the acceptor channel represents the detection of Cy3-Cy5 dimers. At the start of imaging all initial detections are assumed to be attributable to pre-existing Cy3-Cy5 dimers present at the start of the experimental acquisition. Consequently, the first three frames are discarded (frame one due to underexposure) to account for detection of these Cy3-Cy5 dimers. Following this, subsequent detection of new acceptor trajectories can be attributed to the formation of new dimer species during the experiment, from which the rate of dimer formation can be extracted. An exponential decay ensues with a decreasing number of new trajectories detected with time, as predominantly donor, and to a lesser extent acceptor, fluorophores photobleach through the time course of the experiment (Fig. 3a,b). Fitting an exponential to this data enables extrapolation to conditions of zero photobleaching at the y-axis intercept providing the rate of Cy3-Cy5 dimer formation.

k_{on}: Having experimentally measured the rate of formation of Cy3-Cy5-labelled (FRET) dimers as 0.0218 $\mu m^2 s^{-1}$ and with FRET-capable dimers representing 0.268 of the dimeric population, we calculate the rate of formation of all dimer species, k_{on}, as 0.0813 $\mu m^2 s^{-1}$ at a total receptor density of 0.428 μm^{-2}.

k_{off}: With the rate of dimer formation and the equilibrium monomer-dimer distribution at the experimental receptor density, dimer lifetime and k_{off} are determined using equation (S2)

$$k_{off}/k_{on} = [A][B]/[AB] \qquad \text{(Eq. S2)}$$

where [AB] represents dimer density, and, with the interaction being homodimeric, [A] and [B] both represent monomer density, such that

$$k_{off}/k_{on} = [\text{monomer}]^2/[\text{dimer}] \qquad \text{(Eq. S3)}$$

The dimer off rate, k_{off}, is calculated as 0.575 s^{-1}.

Consequently, the half-life of the NTS1 dimer is calculated using equation (S4)

$$t_{1/2} = \ln 2 \ / \ k_{\text{off}} \qquad \text{(Eq. S4)}$$

giving dimer $t_{1/2}$ = 1.205 s. K_a and K_d are calculated by equations (S5), (S6) and (S7).

$$K_a = k_{\text{on}}/k_{\text{off}} \qquad \text{(Eq. S5)}$$

K_a = 0.1413 μm^2.

$$K_d = k_{\text{off}}/k_{\text{on}} \qquad \text{(Eq. S6)}$$

or

$$K_d = [\text{A}][\text{B}]/[\text{AB}] \qquad \text{(Eq. S7)}$$

K_d = 7.0784 μm^{-2}.

Supplementary Note 2 - Quantifying False Positive Dimerisation Detections

We have previously demonstrated in single colour diffraction-limited experiments in droplet interface bilayers that interacting alpha hemolysin monomers co-localise for no longer than 5 ms where oligomerisation does not proceed to the stable heptameric state.[44] The individual monomer-monomer interaction of these membrane proteins is short-lived, despite their ability to ultimately form stable heptameric pores. The reported experiments find that heptamerisation is rare, but occurs rapidly (<5 ms) to produce a small number of stable heptamers in a high density monomeric population, with no intermediate oligomers persisting beyond the 5 ms timescale. Consequently, this benchmark provides strong evidence that the timescales of interaction for NTS1 monomers reported in this work (90+ ms) can reliably be attributed solely to bona fide and stable (although transient) protein-protein interaction and therefore dimerisation.

The contribution of chance (i.e. non-interacting), diffraction-limited co-localised trajectories to observed dimerisation events has been demonstrated to be negligible on these timescales in live cell

single-molecule imaging.[45] In the well-controlled in vitro DIB system reported here we can have greater confidence that measured interactions are driven by molecular interaction alone, by virtue of making measurements in a minimal system with membrane diffusion unhindered by the cytoskeleton or other cellular features. Here we can extend the approach of Hern et al.[45] and experimentally determine the contribution of prolonged chance co-localised events by comparing trajectories in the continuously visualised Cy3 channel in multiple experiments. To this end we combine all measured Cy3-NTS1 trajectories from five separate experimental videos to provide an equivalent trajectory density to that of the non-visualised Cy5-labelled species. Every trajectory was then tested for spatial and temporal coincidence with all trajectories in a sixth Cy3-NTS1 video, serving as a set of donor trajectories. Coincidence was determined at different spatial proximities determining the number of chance co-localisations over different timescales and spatial distances. At a coincidence diameter of 200 nm, approximating to the proximity limit for determining diffraction-limited co-localisation, we find an equivalent to 15.1% of our measured dimer traces would be attributable to the chance co-localisation of two receptors within 200 nm over three or more consecutive frames. It is notable that these represent 3 frame (10.65%) and 4 frame (4.44%) events only, with no longer lasting coincident diffusion events detected (Supplementary Fig. 5). This represents the false positive rate if determination of receptor-receptor interaction were made by diffraction-limited means at our reconstituted receptor density.

In the dimerisation experiments reported here, we use FRET to afford more than an order of magnitude greater spatial resolution in determining interaction compared to diffraction-limited co-localisation and have accepted only interactions persisting for longer than 90 ms (3 frames), providing a stringent threshold in attributing interaction for classification as NTS1 dimers. Since we employ FRET to determine receptor-receptor interaction, two receptors must be within the order of the Förster radius, in this case approximately 5 nm, to be detected as interacting and attributed as a dimer. At this length scale no coincident trajectories persisting for more than 3 frames (the employed cut-off) are observed (Supplementary Fig. 5), demonstrating that chance coincident diffusion makes a negligible contribution to measured dimer trajectories. By plotting the relationship of coincidences persisting for a minimum of three frames against spatial radius defining coincidence, and fitting a decay curve, we estimate a maximum false positive detection rate of 0.04% in our single-molecule FRET experiments,

confirming that co-diffusing, non-interacting, species do not significantly contribute to dimerisation detections in our population of 1,167 measured trajectories. These measurements are in good agreement with analytical solutions for receptor collision which indicate the rarity of co-occupation, even momentarily, of two receptors within a collision radius of each other (of comparable magnitude to the Förster radius). This is borne out by Monte Carlo simulations and the experimentally observed steep reduction in detected dimers with small reduction in receptor density (Supplementary Fig. 4a), as a direct consequence of the decreasing probability of receptor collision.

Supplementary Note 3 - Calculation of collision frequency and estimation of productivity

Dimer formation is a stochastic event initiated by the chance collision of two diffusing receptors in the bilayer. At the single-molecule level concentrations studied, such collisions are relatively infrequent and this is reflected in the frame-to-frame variability in detected dimer arrival events (Fig. 3a,b). With experimental knowledge of receptor diffusion coefficient and receptor density, collision frequency can be estimated by the method of Hardt.[46] Briefly, the mean receptor collision time is calculated by reducing the problem to a model assuming uniformly spaced receptors (sinks) within the membrane, and calculating the average diffusion time for collision with a single diffusing species. The experimental receptor density informs the sink spacing defining a repeating network of 2D cells, each containing one receptor sink. Here, symmetry indicates that the area perimeter can be treated as a reflective boundary owing to the assumption of uniform sink spacing, as all neighbouring cells are nominally identical. Consequently, the average collision time can be calculated considering a single receptor pair of sink and diffusive species within one circular cell with reflective perimeter.[47] In this case, the mean diffusion time of a species with diffusion coefficient, D, to the sink of radius a (defined by the receptor collision radius), within a circular plane of radius b, is given by equation (S8)

$$\mathrm{t} = (b^2/2D)\ln(b/a) \qquad \text{(Eq. S8)}$$

By this method we estimate 0.163 FRET capable collisions s^{-1} μm^{-2}. With an experimentally observed Cy3-Cy5 dimer formation rate of 0.0218 μm^{2} s^{-1} at this receptor concentration, we estimate that 20.15% of collisions result in productive dimerisation.

Supplementary Note 4 - Estimation of error

Our single-molecule FRET experiments provide a time-limited glimpse into the dynamic equilibrium of NTS1 dimerisation. Since we are monitoring short-lived ($t_{1/2}$ = 1.205 s), stochastic events, occurring at low frequency and low concentration (0.0813 μm^2 s^{-1}), measurement variation is expected from video to video, in addition to experimental variation due to differences in reconstitution efficiency between bilayers. To characterise this variance in equilibrium data each video file was analysed individually, in addition to combining all videos for analysis, in accordance with the data analysis procedure detailed above. The error in dissociation constant K_d was determined by fitting to the observed monomer-dimer equilibrium plot generated following individual analysis of each video file. An approximation derived from Hardt[46] (Supplementary Note 3) was used to fit the data, supported by the observed relationship at low receptor densities modelled by Monte Carlo simulation, where receptors are predominantly monomeric and collisions, dictated by area per receptor, determine the dimeric population. For the calculation of error in k_{on}, although the rate of dimerisation depends upon receptor density, since the number of observed events is small, we estimate error of dimer formation rate in all single-molecule experiments. Dimer formation events are plotted as observed against time and fitted with an exponential to account for photobleaching (Supplementary Note 1) and the error on this intercept was determined. Standard error propagation was used to determine the error in k_{off} from these fits. By this method, at a receptor density of 0.43 μm^{-2}, 95% confidence intervals of K_d = [6.70, 12.34] μm^{-2}, k_{on} = [0.072,0.088] μm^2 s^{-1} and k_{off} = [0.45,1.07] s^{-1} were calculated.

Similar observational variance is also found by Monte Carlo simulation. Simulation informed by the experimentally derived kinetic parameters provides further insight beyond the limited temporal window afforded by single-molecule imaging. Supplementary Fig. 4b illustrates the dynamic fluctuation of monomer and dimer subpopulations in a population of 300 receptors, within and beyond the timescale of the experimental observation, illustrating the influence of the arbitrary sampling point of the initiation of imaging. This together with variation in receptor density and the concentration dependency of dimerisation goes some way towards explaining the measured experimental variance. Furthermore, since only the Cy3-Cy5 subpopulation of dimeric species is visualised, temporal fluctuations in this subpopulation are amplified when inferring the total monomeric and dimeric populations. This is illustrated by Monte Carlo simulation and tracking of all dimer label combinations (Supplementary Fig. 4b).

Supplementary Note 5 - Estimation of smFRET distance measurements

The inter-label distance in Cy3-Cy5 NTS1 dimers was estimated from acceptor intensity distributions of both the dimer species and a monomeric doubly labelled mutant (Supplementary Fig. 11). A Cy3-Cy5 Förster radius (R_0) of 54 Å was adopted following previously reported values.[48] For the doubly labelled mutant NTS1 a Cy3-Cy5 distance of r = 1.5 nm was ascribed based on the crystal structure of NTS1.[49] FRET efficiency (E) is therefore defined by equation (S9).

$$E = 1/(1+(r/R_0)^6) \qquad \text{(Eq. S9)}$$

At a distance of 1.5 nm, E = 0.9995 = ~1. Consequently, we assume 100% energy transfer in the case of the doubly labelled mutant, where a median single-molecule acceptor intensity of 146.4 counts is measured (Fig. 2) as a result of donor excitation and energy transfer. Here, we reverse the sensitised acceptor emission approach typically used to estimate distances based on the quantification of donor emission quenched by FRET. Since this is anti-correlated with acceptor emission to define E, we assess the distance-dependent decrease in energy transfer via acceptor intensity decrease compared to that at a known distance corresponding to E = 1. For dimeric Cy3 and Cy5 labelled NTS1 a median single-molecule acceptor intensity of 91.7 counts is measured (Fig. 2), corresponding to 0.63 of the emission observed at ~100% efficient energy transfer of the doubly labelled mutant. Consequently, an energy transfer efficiency of 0.63 is calculated to correspond to an approximate Cy3-Cy5 distance of 5 nm, in close agreement to that obtained by ensemble FRET measurements. Additionally, a sub-population of high acceptor intensity dimers is observed (Fig. 2). Here, the Gaussian centre intensity of 148 counts corresponds closely to the intensity measured in the high efficiency doubly labelled construct with known dye-dye separation of ~1.5 nm. This distance corresponds to the spacing between two neighbouring transmembrane helices in single receptor. A comparable distance may be anticipated between interfacial transmembrane helices of a dimer complex, indicative of a sub-population of dimers with short TM4-4 separation and a larger population with longer TM4-4 distances, approaching the Förster radius for the Cy3-Cy5 label pair. Estimation of TM4-4 separation from FRET efficiency (E) data (Fig. 4) yielded comparable results, with an average dimer TM4-4 Cy3-Cy5 distance of 5.3 nm.

The use of narrow band emission filters, whilst reducing total photon detection, eliminated detectable emission bleed between donor and acceptor channels. Cross-excitation of acceptor by the illumination laser was not detectable at the single-molecule level in the described imaging setup and consequently was considered negligible. It should be noted that donor and acceptor dye orientation also affect energy transfer efficiency. This may be indistinguishable from small changes in distances. However, changes in fluorescent energy transfer are indicative of a conformational change, with large changes in efficiency, as we observe here with the two dimer population states, likely involving significant changes in dye distances due to bona fide conformational changes, given the short linker length.

Supplementary Note 6 - Single-molecule acceptor intensity threshold analysis

Acceptor intensity fluctuation giving rise to two observed dimer acceptor intensity state populations (Fig. 2) was investigated by implementing an intensity threshold crossing algorithm for all acceptor trajectories. The relative contribution of the high- and low-FRET populations was used to inform a conservative threshold to define population switching events (Fig. 4). This was favoured over a more simple mid-point assignment to reduce the probability of assigning false positive events, accepting the implicit trade-off in greater rejection of bona fide population switching events. A threshold of 166.5 counts was assigned (Fig. 4d and Supplementary Fig. 8). Intensities above this threshold represent 42% of high-FRET state spot intensities and just 0.95% of the low-intensity population. Dimer acceptor (Cy5) trajectories were analysed for crossing this defined population threshold, and the dwell-time spent in either high- or low-intensity states prior to either a successive threshold crossing, or trajectory termination by either dimer dissociation or photobleaching, was measured. Cumulative probability plots display the fraction of the observations (on the y-axis) below a given dwell-time given on the x-axis (Fig. 4e). NTS1 dimers were seen to exhibit a greater dwell-time in low-intensity conformation state(s) (Fig. 4e, top panel) than in a high-intensity state (Fig. 4e, bottom panel), in accordance with the relative mixing proportions of the two-component intensity distributions. This is likely indicative of a more stable dimer conformation of lower FRET efficiency, with forays into higher intensity conformations before rapid switching again to a lower intensity conformation (Fig. 4e). However, it should be noted that the high intensity threshold employed to describe high state events will likely over-report high-state to low-state transitions as a result of acceptor intensity fluctuations

within the log-normal distribution of the high-intensity population state. Such a trade-off is unavoidable given the broad nature and overlap of the two population intensity distributions, but is offset by the increased confidence in assigning high-state events. Cumulative lifetime probability of low acceptor intensity states (Fig. 4e, top panel) reveals an apparent divergence of dwell-time in the low-intensity state dependent upon whether this low-state conformation is adopted as the initial (light blue line), mid- (mid blue line), or final (dark blue line) recorded transition of the dimer trajectory. While mid-trajectory low-FRET states show relatively short dwell-times, low-FRET states at the end of trajectories (final states) show relatively long dwell-times. Interestingly, initial low-FRET states show a bimodal cumulative probability curve that first follows the "mid-trajectory" curve, but also shows significant probability to observe longer dwell-times as found in the final low-FRET states. If the low-FRET state represented one dimer species, the observed dwell-time would not be expected to depend on whether the corresponding dimer species was formed at the beginning of a trajectory or after a conversion to a high-FRET dimer species. Thus, the observed differences in low-FRET state dwell-time could be indicative of the presence of two or more dimer conformations affording a similar Cy3-Cy5 distance (and thus observed Cy5 intensity); that is, two or more conformations displaying a differing propensity of formation or differing stability (giving rise to different dwell-time probabilities), yet with a capacity to interconvert via a high-FRET state intermediate during the lifetime of the transient dimer. Further experimentation would be required to elucidate the exact nature of the phenomena underpinning these interesting observations.

Single-molecule acceptor intensity distribution together with ensemble energy transfer measurements reveals multiple dimer conformations, with single-molecule acceptor intensity threshold analysis evidencing likely interconversion between high- and low-acceptor intensity conformations. Further analysis of single-molecule dimer trajectory lifetime measurements is also consistent with the presence of multiple FRET capable conformations displaying differing stabilities. An atypical acceptor lifetime distribution is measured, consistent with both rapid and longer decay processes with superimposed photobleaching (Supplementary Fig. 11). Direct observation of acceptor and donor trajectory lifetime, together with the average dimer lifetime (1.7 s) calculated from measured single-molecule data, show that acceptor trajectory observation is frequently limited by photobleaching. However, the acceptor lifetime histogram also shows an otherwise unanticipated initial increase in

probability towards longer trajectory observations, to a probability maximum at 150 ms. (Supplementary Fig. 11c) A monotonically decreasing lifetime probability is expected for photobleaching-limited observations (as per donor histogram, Supplementary Fig. 11b). Acceptor lifetime probability is observed to be non-monotonic, with a further, more rapid, decay process contributing to a lifetime peak at 150 ms. At this timescale we can rule out incidental collisions and the possibility of any systematic error due to misdetection of acceptor spots, or inaccurate trajectory linking (which would display a monotonically decreasing probability of successively longer tracks). The observation of longer (180+ ms) trajectories displaying a decaying observation probability, on the order of donor photobleaching, is consistent with the simultaneous presence of longer surviving dimer species where photobleaching largely limits observation times.

Supplementary Note 7 – Reconstitution orientation

In the presented work we do not have experimental control over the reconstitution orientation of NTS1 in the artificial membrane. NTS1 reconstitution in liposomes using the same methods as used in this study has previously been reported by our laboratory to yield symmetric reconstitution with ~50/50 distribution of both orientations.[43] The reconstitution orientation in the droplet interface bilayers (DIBs) used in our single-molecule work is unknown. This leads to a number of theoretically possible scenarios:

1) Reconstitution is asymmetric (i.e. all monomers are inserted in the same orientation);
2) Reconstitution is symmetric (i.e. monomers are inserted in random orientations), and antiparallel dimers do not from;
3) Reconstitution is symmetric, and antiparallel dimers can form.

In scenario #1 our analysis would not be affected.

In scenario #2, our reconstitution efficiency would effectively be off by a factor 2, since each receptor will only be able to interact with half of the receptors present. This would mean that our measured/derived parameters would (only) be underestimated by a factor of 2, specifically: the dimer half-life, $t_{1/2}$=1.2 s, (95% CI [0.65,1.54] s), and the K_d=7.1 μm^{-2} (95% CI [6.7,12.3] μm^{-2}). The rate of

dimer formation, k_{on}=0.081 μm^{-2}s^{-1} (95% CI [0.072,0.088] μm^{-2}s^{-1}) is measured directly from the experiment (as detailed in Supplementary Note 1) and would not be affected.

In scenario #3, in theory, any measured parameter would represent a population-weighted average of the parallel and the non-physiological antiparallel dimers. However, we would not expect to observe a large part of any hypothetical antiparallel dimers via FRET because the inter-label distance would be too large to give rise to efficient energy transfer for most hypothetical antiparallel dimer configurations.

Whilst, we cannot definitively exclude any of the three aforementioned scenarios, the observed dimer half-life (1.2 s) is consistent with previous reports on other GPCR dimers (~0.1-5 s).[45,50,51]
Additionally, the observed TM4-4 distance of ~5.0 nm for the low FRET state and shorter distance (~1.5 nm) for the high-FRET state are too short to both originate from antiparallel dimers, for which we would expect a minimal distance of ~4-7 nm. Thus, neither the high-FRET state observed in smFRET, nor the short distances observed by DEER, could originate from an antiparallel dimer. Furthermore, the smFRET threshold crossing analysis (presented in Fig. 4) showed that individual dimers sample both the low- and high-FRET states, i.e. show interconversion between these states. Both these states must thus originate from a parallel dimer to be able to interconvert, validating our conclusion that the physiologically relevant dimer samples multiple interfaces.
In addition, the relative proportion of low- and high-FRET states identified from the FRET efficiency histogram (80/20, Fig. 4c) is very similar to that identified from the dimer acceptor fluorescence intensity distribution (84/16, Fig. 2a). Notably, FRET efficiencies were only calculated from a subset of long-lived dimers (to be able to extract meaningful acceptor trajectories) and are thus more likely to include contributions from the high-FRET state. Thus, the FRET efficiency histogram is biased towards trajectories containing transitions between low- and high-FRET states, which, as argued above, cannot originate from antiparallel dimers. The dimer acceptor fluorescence intensity histogram does not have this bias and also includes shorter traces that are more likely to only sample the low FRET states. If hypothetical antiparallel dimers with long, but observable inter-label distances were present in significant proportions, this would be reflected in a much higher low FRET population in the acceptor fluorescence intensity distribution histogram compared to the FRET efficiency histogram, which we do not observe.

Taken collectively, while we cannot exclude the possibility of random insertion in the bilayer, and the formation of antiparallel dimers with the data at hand, we consider it reasonable to conclude that any effect must be modest.

Supplementary Note 8 – Microscale thermophoresis: ligand and G protein binding of NTS1 mutants

Native-like activity of cysteine mutants in detergent was verified by ligand and G protein binding experiments using MST. Cysteine-depleted NTS1 was shown to bind fluorescent NT (NT-Cy5) with an affinity similar to that observed for WT, giving affinity dissociation constants $K_d \leq 2$ nM and $K_d \leq 10$ nM, respectively (Supplementary Fig. 2). K_d values are given as an upper limit, as the symmetric error distribution assumed by the fitting procedure would give rise to negative values, but the K_d can thus also be lower. These values are in good agreement with previously reported low-nanomolar (1-2 nM) values.[52] Radio-ligand binding assays[53] showed that spin labelling with MTSL also did not affect ligand-binding, with 100 ± 20% activity retained (n = 6, for TM5-6 cysteine mutants). G protein ($G\alpha_{i1}$) binding to cysteine mutants labelled with Alexa Fluor 488 on TM4 (T186C) or TM6 (V307C), showed slightly lower, but comparable binding affinity to WT-NTS1, labelled using the native cysteines (Supplementary Fig. 3). Although it cannot be excluded that the intracellular position of the relatively large fluorophores in the mutants interferes with the G protein interaction, addition of GTPγS (non-hydrolysable analogue of GTP) lowered the binding affinity by at least one order of magnitude (Supplementary Fig. 3b,d,f), suggesting G protein does bind specifically to both the WT and mutant receptors. Similar affinity values for the NTS1-G protein interaction have been previously reported; $G\alpha_{i1}$ was shown to bind to WT-NTS1 in nanodiscs of various lipid compositions with a K_d of ~100-600 nM[54], and in radioactivity assays detergent-solubilised NTS1 was shown to catalyses GTP exchange at the $G\alpha_q\beta_1\gamma_2$ heterotrimer with an apparent EC_{50} of ~145 nM.[55]

Supplementary Note 9 – Ensemble FRET data analysis

For FRET samples (containing both donor and acceptor), and acceptor-only samples emission spectra with excitation at the donor (490 nm) and acceptor (555 nm) wavelength were recorded (over 495-600, and 560-600 nm, respectively). For donor-only samples only the emission spectrum with excitation at the donor wavelength was recorded. A background sample consisting of empty liposomes was also prepared and measured to correct for any background fluorescence and

scattering effects in the sample spectra. The FRET data was processed as described by Goddard et al.[56] First, the background spectrum (empty liposomes) was subtracted from all spectra. Spectra were smoothed using Savitzky-Golay filtering (10 nm window, 2nd order polynomial) using OriginPro 8.5 to minimise noise artefacts in the determination of spectral maxima. Then, the FRET spectrum (mixed sample at donor excitation) was corrected for bleedthrough by subtracting the donor-only spectrum, scaled to the donor peak in the FRET spectrum, and the uncorrected FRET ratio (A) was then calculated

$$A = F_{\mathrm{ex:D}}^{\mathrm{em:A}} / F_{\mathrm{ex:A}}^{\mathrm{em:A}} \qquad \text{(Eq. S10)}$$

where $F_{\mathrm{ex:D}}^{\mathrm{em:A}}$ is the fluorescence intensity of bleedthrough-corrected FRET spectrum at the acceptor emission maximum, and $F_{\mathrm{ex:A}}^{\mathrm{em:A}}$ the intensity of the FRET sample at the acceptor maximum upon acceptor excitation. The crosstalk ratio was determined from the acceptor-only sample (ratio A_0),

$$A_0 = F_{\mathrm{ex:D}}^{\mathrm{em:A}} / F_{\mathrm{ex:A}}^{\mathrm{em:A}} \qquad \text{(Eq. S11)}$$

where $F_{\mathrm{ex:D}}^{\mathrm{em:A}}$ and $F_{\mathrm{ex:A}}^{\mathrm{em:A}}$ are the fluorescence intensity for the acceptor-only sample at the acceptor emission maximum upon excitation at the donor or acceptor wavelength, respectively. The bleedthrough-corrected FRET ratio (FR) was calculated

$$\mathrm{FR} = A / A_0 \qquad \text{(Eq. S12)}$$

from which the apparent FRET efficiency (E_{app}) was calculated,

$$E_{\mathrm{app}} = (\mathrm{FR} - 1) \frac{\varepsilon_{\mathrm{A}}^{490}}{\varepsilon_{\mathrm{D}}^{490}} \qquad \text{(Eq. S13)}$$

correcting for the extinction coefficient ratio of the acceptor (ε_{A}) and the donor (ε_{D}) fluorophores at the donor excitation wavelength used, which in the case for Alexa Fluor 555 and 488, corresponds to

15.2% and 81.7% of the maximum extinction coefficients, respectively. The apparent FRET efficiency was further corrected for the donor-to-acceptor ratio (r_{DA}) in the FRET samples (see Methods).

Supplementary Note 10 - Estimation of lipid-to-protein ratio for ensemble experiments

Discontinuous sucrose-density gradients (5-35% w/v sucrose in 50 mM Tris-HCl pH 7.4, 50 mM NaCl, 1 mM EDTA, with 5% sucrose steps) were run of liposome-reconstituted NTS1 to estimate the lipid-to-protein ratio after reconstitution. NTS1 proteoliposomes were layered on top of the gradients, which were centrifuged overnight in a swing-out rotor (SW41, Beckman Coultier) at 28,500 rpm (~100,000 *g*). The sucrose gradient was then fractionated and the presence of reconstituted receptor verified by SDS-PAGE analysis. The position of the proteoliposome band on the gradient was used to estimate the density and thus the lipid-to-protein (L:P) ratio of the samples as described by Goddard et al.[42] The observed density for three separate gradients for the FRET samples, and four for the DEER samples was averaged. The FRET samples ran as a single band, while for the DEER samples a second band corresponding to empty liposomes was observed (as verified by SDS-nPAGE analysis). The final L:P ratios were substantially lower than the initial ratios used in the reconstitution, with estimated ratios of 840±20 (6,000:1 initial) and 410±10 (1,500:1 initial) for the FRET and DEER samples, respectively (Supplementary Table 6). The origin hereof is unclear, but it has been observed previously for detergent-mediated reconstitution of NTS1[43], and could in part be due to absorption of lipids by the hydrophobic Bio-Beads used in the reconstitution procedure[57], and due to not all liposomes incorporating protein (as a band corresponding to empty liposomes was seen on the sucrose gradients of NTS1 DEER samples). Assuming the vesicle radius to be 500 Å (as unilamellar liposomes were created by extrusion through 100 nm filters), and the internal radius as 460 Å (subtracting a bilayer thickness of 40 Å), the total surface area of the liposome bilayer is estimated at 5.8x10^{6} Å^{2} (including both surfaces of the bilayer). Using the total area (A_T), the number of receptor molecules (n_R) per vesicle can be estimated from

$$A_T = n_L A_L + 2 n_R A_R \qquad \text{(Eq. S14)}$$

and with lipid-to-protein ratio

$$r = n_L / n_R \qquad \text{(Eq. S15)}$$

it follows that

$$n_R = A_T/(rA_L + 2A_R) \quad \text{(Eq. S16)}$$

where A_R and A_L are the surface area of the receptor and the lipid molecules, which were taken to be 71.1 Å^2 (area of a POPC molecule[58]) and 1350 Å^2 (protein size estimated from crystal structure ~30x45 Å[5]), and n_L is the number of lipids. Thus, the number of receptors per vesicle was estimated to be 90 ± 20 and 180 ± 40, corresponding to a receptor density of 1.6 ± 0.3 x10^3 and 3.1 ± 0.6 x10^3 molecules per μm^2 for the FRET and DEER samples, respectively (Supplementary Table 6). This is similar to receptor densities reported in the literature for cell studies based on heterologous over-expression of the β_2-adrenergic receptor[59], but is similarly well above expected physiological densities of ~6000 copies per cell (~2 molecules per μm^2).[50] However, the BRET efficiency due to β_2-adrenergic receptor dimerisation in the cell-based study was constant over the same density range as used here, suggesting that bystander BRET (or FRET) due to crowding is minimal under these conditions.[59] Indeed, a previous FRET study on NTS1 under similar conditions did not see dependence of the apparent FRET efficiency on the lipid-to-protein ratio (at this order of magnitude).[43]

Supplementary references

1. Leptihn, S. *et al.* Constructing droplet interface bilayers from the contact of aqueous droplets in oil. *Nat. Protoc.* **8,** 1048–1057 (2013).
2. Polyhach, Y., Bordignon, E. & Jeschke, G. Rotamer libraries of spin labelled cysteines for protein studies. *Phys. Chem. Chem. Phys.* **13,** 2356–2366 (2011).
3. Seidel, S. A. I. *et al.* Microscale thermophoresis quantifies biomolecular interactions under previously challenging conditions. *Methods* **59,** 301–315 (2013).
4. Jeschke, G. *et al.* DeerAnalysis2006 - a comprehensive software package for analyzing pulsed ELDOR data. *Appl. Magn. Reson.* **30,** 473–498 (2006).
5. Egloff, P. *et al.* Structure of signaling-competent neurotensin receptor 1 obtained by directed evolution in Escherichia coli. *Proc. Natl. Acad. Sci. USA* **111,** E655–E662 (2014).
6. Huang, J., Chen, S., Zhang, J. J. & Huang, X.-Y. Crystal structure of oligomeric β_1-adrenergic G protein-coupled receptors in ligand-free basal state. *Nat. Struct. Mol. Biol.* **20,** 419–425 (2013).
7. Wu, B. *et al.* Structures of the CXCR4 chemokine GPCR with small-molecule and cyclic peptide antagonists. *Science* **330,** 1066–1071 (2010).
8. Shimamura, T. *et al.* Structure of the human histamine H_1 receptor complex with doxepin. *Nature* **475,** 65–70 (2011).
9. Wu, H. *et al.* Structure of the human κ-opioid receptor in complex with JDTic. *Nature* **485,** 327–332 (2012).
10. Manglik, A. *et al.* Crystal structure of the µ-opioid receptor bound to a morphinan antagonist. *Nature* **485,** 321–326 (2012).
11. Duhovny, D., Nussinov, R. & Wolfson, H. J. *Algorithms in Bioinformatics: WABI 2002. Lecture notes in computer science,* vol 2452, 185–200 (Springer, Berlin, Heidelberg, 2002).
12. Schneidman-Duhovny, D., Inbar, Y., Nussinov, R. & Wolfson, H. J. PatchDock and SymmDock: Servers for rigid and symmetric docking. *Nucleic Acids Res.* **33,** 363–367 (2005).
13. Hebert, T. E., Moffett, S., Morello, J.-P., Loisel, T. P. & Bichet, D. G. A peptide derived from a β_2-adrenergic receptor transmembrane domain inhibits both receptor dimerization and activation. *J. Biol. Chem.* **271,** 16384–16392 (1996).
14. Cherezov, V. *et al.* High-resolution crystal structure of an engineered human β_2-adrenergic G protein-coupled receptor. *Science* **318,** 1258–1265 (2007).
15. Fung, J. J. *et al.* Ligand-regulated oligomerization of β_2-adrenoceptors in a model lipid bilayer. *EMBO J.* **28,** 3315–3328 (2009).
16. Suda, K., Filipek, S., Palczewski, K., Engel, A. & Fotiadis, D. The supramolecular structure of the GPCR rhodopsin in solution and native disc membranes. *Mol. Membr. Biol.* **21,** 435–446 (2004).
17. Kota, P., Reeves, P. J., Rajbhandary, U. L. & Khorana, H. G. Opsin is present as dimers in COS1 cells: identification of amino acids at the dimeric interface. *Proc. Natl. Acad. Sci. USA* **103,** 3054–3059 (2006).
18. Zeng, F.-Y. & Wess, J. Identification and Molecular Characterization of m3 Muscarinic

Receptor Dimers. *J. Biol. Chem.* **274,** 19487–19497 (1999).

19. McMillin, S. M., Heusel, M., Liu, T., Costanzi, S. & Wess, J. Structural basis of M_3 muscarinic receptor dimer/oligomer formation. *J. Biol. Chem.* **286,** 28584–28598 (2011).
20. Hu, J. *et al.* Structural aspects of M_3 muscarinic acetylcholine receptor dimer formation and activation. *FASEB J.* **26,** 604–616 (2012).
21. Ng, G. Y. *et al.* Dopamine D2 receptor dimers and receptor-blocking peptides. *Biochem. Biophys. Res. Commun.* **227,** 200–204 (1996).
22. Lee, S. P., O'Dowd, B. F., Rajaram, R. D., Nguyen, T. & George, S. R. D2 dopamine receptor homodimerization is mediated by multiple sites of interaction, including an intermolecular interaction involving transmembrane domain 4. *Biochemistry* **42,** 11023–11031 (2003).
23. Guo, W., Shi, L. & Javitch, J. A. The fourth transmembrane segment forms the interface of the dopamine D2 receptor homodimer. *J. Biol. Chem.* **278,** 4385–4388 (2003).
24. Guo, W., Shi, L., Filizola, M., Weinstein, H. & Javitch, J. A. Crosstalk in G protein-coupled receptors: changes at the transmembrane homodimer interface determine activation. *Proc. Natl. Acad. Sci.* **102,** 17495–17500 (2005).
25. Guo, W. *et al.* Dopamine D2 receptors form higher order oligomers at physiological expression levels. *EMBO J.* **27,** 2293–2304 (2008).
26. Marsango, S., Caltabiano, G., Pou, C., Varela Liste, M. J. & Milligan, G. Analysis of Human Dopamine D_3 Receptor Quaternary Structure. *J. Biol. Chem.* **290,** 15146-15162 (2015).
27. Benkirane, M., Jin, D.-Y., Chun, R. F., Koup, R. A. & Jeang, K.-T. Mechanism of Transdominant Inhibition of CCR5-mediated HIV-1 Infection by ccr5Δ32. *J. Biol. Chem.* **272,** 30603–30606 (1997).
28. Hernanz-Falcón, P. *et al.* Identification of amino acid residues crucial for chemokine receptor dimerization. *Nat. Immunol.* **5,** 216–223 (2004).
29. Wang, J., He, L., Combs, C. A., Roderiquez, G. & Norcross, M. A. Dimerization of CXCR4 in living malignant cells: control of cell migration by a synthetic peptide that reduces homologous CXCR4 interactions. *Mol. Cancer Ther.* **5,** 2474–2483 (2006).
30. Michineau, S., Alhenc-Gelas, F. & Rajerison, R. M. Human bradykinin B2 receptor sialylation and N-glycosylation participate with disulfide bonding in surface receptor dimerization. *Biochemistry* **45,** 2699–2707 (2006).
31. AbdAlla, S., Zaki, E., Lother, H. & Quitterer, U. Involvement of the amino terminus of the B_2 receptor in agonist-induced receptor dimerization. *J. Biol. Chem.* **274,** 26079–26084 (1999).
32. Overton, M. C. & Blumer, K. J. The extracellular N-terminal domain and transmembrane domains 1 and 2 mediate oligomerization of a yeast G protein-coupled receptor. *J. Biol. Chem.* **277,** 41463–41472 (2002).
33. Wang, H. X. & Konopka, J. B. Identification of amino acids at two dimer interface regions of the α-factor receptor (Ste2). *Biochemistry* **48,** 7132–7139 (2009).
34. Uddin, M. S., Kim, H., Deyo, A., Naider, F. & Becker, J. M. Identification of residues involved in homodimer formation located within a β-strand region of the N-terminus of a yeast G protein-coupled receptor. *J. Recept. Signal Transduct. Res.* **32,** 65–75 (2012).

35. Cvejic, S. & Devi, L. A. Dimerization of the δ opioid receptor: Implication for a role in receptor internalisation. *J. Biol. Chem.* **272,** 26959–26964 (1997).
36. Casciari, D., Dell'Orco, D. & Fanelli, F. Homodimerization of neurotensin 1 receptor involves helices 1, 2, and 4: insights from quaternary structure predictions and dimerization free energy estimations. *J. Chem. Inf. Model.* **48,** 1669–1678 (2008).
37. Mancia, F., Assur, Z., Herman, A. G., Siegel, R. & Hendrickson, W. A. Ligand sensitivity in dimeric associations of the serotonin 5HT2c receptor. *EMBO Rep.* **9,** 363–369 (2008).
38. Thévenin, D., Lazarova, T., Roberts, M. F. & Robinson, C. R. Oligomerization of the fifth transmembrane domain from the adenosine A_{2A} receptor. *Protein Sci.* **14,** 2177–2186 (2005).
39. Lopez-Gimenez, J. F., Canals, M., Pediani, J. D. & Milligan, G. The α_{1b}-adrenoceptor exists as a higher-order oligomer: effective oligomerization is required for receptor maturation, surface delivery, and function. *Mol. Pharmacol.* **71,** 1015–1029 (2007).
40. Banères, J.-L. & Parello, J. Structure-based analysis of GPCR function: evidence for a novel pentameric assembly between the dimeric leukotriene B_4 receptor BLT1 and the G-protein. *J. Mol. Biol.* **329,** 815–829 (2003).
41. Burnham, K. P., Anderson, D. R. & Huyvaert, K. P. AIC model selection and multimodel inference in behavioral ecology: some background, observations, and comparisons. *Behav. Ecol. Sociol.* **65,** 23–35 (2011).
42. Goddard, A. D., Dijkman, P. M., Adamson, R. J., dos Reis, R. I. & Watts, A. Reconstitution of Membrane Proteins: A GPCR as an Example. *Methods in enzymology* **556**, 405-424 (2015).
43. Harding, P. J. *et al.* Constitutive dimerization of the G-protein coupled receptor, neurotensin receptor 1, reconstituted into phospholipid bilayers. *Biophys. J.* **96,** 964–973 (2009).
44. Thompson, J. R., Cronin, B., Bayley, H. & Wallace, M. I. Rapid Assembly of a Multimeric Membrane Protein Pore. *Biophys. J.* **101,** 2679–2683 (2011).
45. Hern, J. A. *et al.* Formation and dissociation of M1 muscarinic receptor dimers seen by total internal reflection fluorescence imaging of single molecules. *Proc. Natl. Acad. Sci. USA* **107,** 2693–2698 (2010).
46. Hardt, S. L. Rates of diffusion controlled reactions in one, two and three dimensions. *Biophys. Chem.* **10,** 239–243 (1979).
47. Axelrod, D. Lateral motion of membrane proteins and biological function. *J. Membrane Biol.* **75,** 1–10 (1983).
48. Ha, T. *et al.* Initiation and re-initiation of DNA unwinding by the *Escherichia coli* Rep helicase. *Nature* **419,** 638–641 (2002).
49. White, J. F. *et al.* Structure of the agonist-bound neurotensin receptor. *Nature* **490,** 508–513 (2012).
50. Kasai, R. S. *et al.* Full characterization of GPCR monomer-dimer dynamic equilibrium by single molecule imaging. *J. Cell Biol.* **192,** 463–480 (2011).
51. Calebiro, D. *et al.* Single-molecule analysis of fluorescently labeled G-protein-coupled receptors reveals complexes with distinct dynamics and organization. *Proc. Natl. Acad. Sci. USA* **110,** 743–748 (2013).

52. Fang, Y., Hong, Y., Webb, B. & Lahiri, J. Applications of Biomembranes in Drug Discovery. *MRS Bull.* **31,** 541–545 (2006).
53. Harding, P. J. *et al.* Neurotensin receptor type 1: *Escherichia coli* expression, purification, characterization and biophysical study. *Biochem. Soc. Trans.* **35,** 760–763 (2007).
54. Dijkman, P. M. & Watts, A. Lipid modulation of early G protein-coupled receptor signalling events. *Biochim. Biophys. Acta - Biomembr.* **1848,** 2889–2897 (2015).
55. White, J. F. *et al.* Dimerization of the class A G protein-coupled neurotensin receptor NTS1 alters G protein interaction. *Proc. Natl. Acad. Sci. USA* **104,** 12199–12204 (2007).
56. Goddard, A. D., Dijkman, P. M., Adamson, R. J. & Watts, A. Lipid-Dependent GPCR Dimerization. *Methods Cell Biol.* **117,** 341–357 (2013).
57. Rigaud, J.-L. *et al.* Bio-Beads: an efficient strategy for two-dimensional crystallization of membrane proteins. *J. Struct. Biol.* **118,** 226–235 (1997).
58. Small, D. M. Phase equilibria and structure of dry and hydrated egg lecithin. *J. Lipid Res.* **8,** 551–557 (1967).
59. Mercier, J.-F., Salahpour, A., Angers, S., Breit, A. & Bouvier, M. Quantitative assessment of β_1- and β_2-adrenergic receptor homo- and heterodimerization by bioluminescence resonance energy transfer. *J. Biol. Chem.* **277,** 44925–44931 (2002).
