# Supplementary material for: Dynamic tuneable G protein-coupled receptor monomer-dimer populations
Source: Nat Commun. 2018 Apr 27;9:1710. doi: 10.1038/s41467-018-03727-6 (PMC5923235; doi:10.1038/s41467-018-03727-6)
Supplement: Supplementary file 3 — Description of Additional Supplementary Information(DOCX 13 kb) [file 41467_2018_3727_MOESM3_ESM.docx]

**Description of Additional Supplementary Files**

File Name: Supplementary Movie 1

Description: Single-molecule FRET overlaid donor (green) and acceptor (red) video,

real time (30 ms per frame).

File Name: Supplementary Movie 2

Description: Single-molecule FRET overlaid donor (green) and acceptor (red) illustrating

FRET initiation as a result of dimer formation as illustrated in Fig. 1 montage, real time (30 ms per frame).

File Name: Supplementary Movie 3

Description: Single-molecule FRET overlaid donor (green) and acceptor (red) illustrating

dimer FRET termination as illustrated in Fig. 1 montage, real time (30 ms per frame).

File Name: Supplementary Movie 4

Description: Progression of a Monte Carlo simulation at single-molecule receptor

density with panels (l-r) showing; i) all simulated molecular trajectories; ii) relative proportion of Cy3 monomers, Cy5 monomers, FRET capable dimers (Cy3-Cy5), C3-Cy3 dimers and Cy5-Cy5 dimers; iii) simulated donor channel and iv) simulated acceptor channel. Simulated time period of 6 s.

File Name: Supplementary Movie 5

Description: Single-molecule FRET overlaid donor (green) and acceptor (red) illustrating

donor and acceptor intensity fluctuation within a dimeric FRET trajectory, real time (30 ms per frame). Note relative display contrast of red and green channels is adjusted compared to Supplementary Movies 1-3.
